# The Spatial Properties of Radical Environmental Organizations in the UK: Do or Die!

**DOI:** 10.1371/journal.pone.0166609

**Published:** 2016-11-29

**Authors:** Zack W. Almquist, Benjamin E. Bagozzi

**Affiliations:** 1 Department of Sociology, School of Statistics and MPC, University of Minnesota, Twin Cities, MN, United States of America; 2 Department of Political Science and International Relations, University of Delaware, Newark, DE, United States of America; Universidad de Alicante, ITALY

## Abstract

Radical environmental groups and their members have a wide and varied agenda which often encompasses both local and global issues. In their efforts to call attention to environmental problems, communicate with like-minded groups, and mobilize support for their activities, radical environmental organizations also produce an enormous amount of text, which can be used to estimate the complex communications and task-based networks that underlie these organizations. Moreover, the tactics employed to garnish attention for these groups’ agenda can range from peaceful activities such as information dissemination to violent activities such as fire-bombing buildings. To obtain these varied objectives, radical environmental organizations must harness their networks, which have an important spatial component that structures their ability to communicate, coordinate and act on any given agenda item. Here, we analyze a network built from communications and information provided by the semi-annual “Do or Die” (DoD) magazine published in the UK over a 10 year period in the late 1990s and early 2000s. We first employ structural topic model methods to discover violent and nonviolent actors within the larger environmental community. Using this designation, we then compare the spatial structure of these groups, finding that violent groups are especially likely to engage in coordination and/or communication if they are sufficiently close, but exhibit a quickly decreasing probability of interaction over even a few kilometers. Further, violent and nonviolent groups each have a higher probability of coordination with their own group than across groups over even short distances. In these respects, we see that violent groups are especially local in their organization and that their geographic reach is likely very limited. This suggests that nonviolent environmental groups seek each other out over both large and short distances for communication and coordination, but violent groups tend to be highly localized.

## Introduction

Radical environmental groups have a wide and varied agenda which often encompasses both local and global issues, as well as violent and nonviolent tactics. Importantly in these regards, in the analysis to follow, we consider property destruction to be a form of violence. Indeed, the tactics employed by such groups can range from peaceful efforts to garner attention for their agenda, such as information dissemination, to more violent activities, such as fire-bombing buildings. Yet, we lack a complete understanding of the varied tactics employed by radical environmental groups—and of these groups’ interactions with one another—given the generally clandestine nature of their activities. We address these deficiencies by noting that, in their efforts to call attention to environmental problems, communicate with like-minded groups, and mobilize support for their activities, radical environmental organizations also produce an enormous amount of text, which can be used to estimate the complex communications and task-based networks that underlie these organizations. Here, we analyze and extend a radical environmental network built from communications and information provided by the semi-annual “Do or Die” (DoD) magazine published in the UK over a 10 year period in the late 1990s and early 2000s [1] so as to offer novel insights into the tactics, and spatial behaviors, of radical environmental groups.

We begin by first extending the network in Almquist and Bagozzi [1] to unpack its spatial distribution through spatial network models. Alongside this network analysis, we employ structural topic models (STMs) [2] of the DoD texts to discover and objectively classify the groups within this network as violent and nonviolent group-actors. After combining these two learned sets of group features, we explore the varied network properties of violent and nonviolent radical environmental groups, and compare their spatial structures. In doing so, we find that both violent and nonviolent groups are more likely to engage in coordination/communication if they are sufficiently close, but exhibit a quickly decreasing probability of interaction over even a few kilometers. We also find that, over a given distance, both groups have a higher probability of coordination/communication with their own group-type than across group-type. Furthermore, in each of these respects, we see that violent groups are especially local in their organization and that their geographic reach is likely very limited.

This analysis demonstrates that there is a spatially-driven homophilious relationship within the violent and nonviolent groups included in our sample and that there is a strong spatial component to their interaction. Our finding that—over any city length distances —both group types have a higher probability of coordination with their own group than across group further implies that violent and nonviolent environmental groups disproportionately seek out like minded groups over moderate-to-large distances. This divergence is consistent with general theories of attribute-based segregation within social networks [3], as well as with anecdotal accounts of the tensions associated with cross-group collaboration when differing perspectives exist among environmental groups [4, 5]. By contrast, our finding that sufficiently proximate violent and nonviolent groups are relatively more likely to cross-collaborate is consistent with extant studies of UK environmental groups, which note for example that among environmental groups in southeast London during this same period, radical and non-radical environmental groups coordinated closely with one another [6].

Beyond these specific findings, this paper offers a number of additional novel contributions. While past studies of radical groups have examined the group-level traits that may distinguish between violent and nonviolent groups (and actions) [7], the present study, to our knowledge, is the first to explicitly compare and contrast the spatial network configurations of violent and nonviolent radical groups—environmental or otherwise. In doing so, we find that spatial proximity remains critical to radical environmental group interactions—and especially to violent environmental groups’ interactions. This finding contradicts the emerging narrative surrounding violent extremist groups, which largely characterizes such groups in modern times as spatially unconstrained and geographically decentralized [8–12]. In this respect, our work follows more recent, sociospatial analyses of Islamic terrorist networks [13] in demonstrating that for networks of violent radicals, local geographic factors remain crucial.

The remainder of this paper is laid out in the following manner: (1) background and research hypothesis, (2) data, (3) social network and spatial network models, (4) results, and (5) discussion and summary.

## Background

Radical environmental groups —which include organizations such as Earth First! (EF!), the Earth Liberation Front (ELF), and the Animal Liberation Front (ALF)—developed in response to growing dissatisfaction with mainstream environmental action in the western world from the 1970-80s. In the ensuing years, these groups have had significant impact upon environmental politics through a hard-line stance towards ecocentrism and strong critiques of mainstream conservationist groups. To achieve these aims, radical environmental groups often stratify between violent acts such as: illegal or controversial protest tactics by environmental radicals–including ecotage, e.g., illegal property destruction for ecological purposes. protest camps (the organized occupation of physical spaces to protest, call attention to, and/or obstruct an activity deemed harmful to the environment or a related issue-area), (nonviolent) direct action (direct action involves an organized activity that is aimed to call attention to, disrupt, or change a behavior considered environmentally unsustainable); and nonviolent acts such as peaceful protests, information gathering, media engagement, and governmental lobbying [14]. The more violent actions of eco-sabotage (e.g., use and threats of violence and property destruction) have often been compared to domestic terrorism [15–17].

Within the UK specifically, the radical environmental movement largely coalesced in early 1990s with the formation of the UK EF! movement. In the beginning, EF! UK drew heavily upon the influence of the previously established US EF! organization—as well as the UK peace movement and earlier UK environmental groups—in pursuing its direct actions. These initial actions were protest-oriented, and included, for example, efforts to establish a peaceful blockade of the Dungeness nuclear power plant and to disrupt the importation of timber into the UK [1]. EF!’s tactics then branched out into larger scale efforts towards mass action, anti-roads protests (in turn spurning new radical environmental groups such as Reclaim the Streets), and later, into additional issue areas such as opposition to airport construction and housing on greenfield sites [18]. By the mid 1990s, the UK EF! movement’s strategies of nonviolent direct action were also increasingly paired with tactics of active sabotage, as both moderate and militant activist groups came to coexist within the broader UK EF! movement [18]. Alongside these evolving strategies, the local groups associated with the EF! UK movement, as well as related radical UK groups, networked extensively with one another through a variety of radical environmentalist publications, including SchNEWS and DoD [1]. The UK EF! movement, and a number of additional radical groups that arose from it remain active to this day, albeit with less prominence.

Given the central position of radical (UK) environmental groups in contemporary environmental politics and society, as well as the high-profile nature of the actions undertaken by these groups, radical environmental organizations occupy a prominent space within studies of social movements, environmental politics, terrorism, and social science more generally. Nevertheless, empirical research on radical environmental groups has been severely constrained by a lack of sufficient high-quality data containing information on the strategies, linkages, and agendas of these groups. This limitation is unsurprising, considering that many radical environmental movements are short lived, or are highly fluid in their ideology, strategies, and membership [19]. Moreover, the ideology of environmental groups, the illegal and/or anti-government protest tactics that they often favor, and past counter-movement efforts led by government or industry actors, have together given environmental groups the incentives to conceal, obfuscate, and misrepresent their membership and ties with other groups [20, 21]. As a consequence of these tendencies, research on environmental action has been largely limited to qualitative case studies and small *N* research. While we find these studies to be insightful, we believe that a more systematic quantitative analysis of environmental groups could serve as a useful compliment to these existing approaches.

## Research Hypothesis

We propose that the mechanism for interaction between violent and nonviolent organizations within the radical environmental community will be markedly different. We expect that violent actors will be engaged in large-scale coordination and active in communication across the UK. Following the work in the political science and social movements literatures [9, 11, 22, 23], we believe that violent and extremist groups will be run by a widely networked and geographically unconstrained set of elite actors with a large scale agenda. Alternatively, we expect that nonviolent groups will be highly localized and oriented towards geographically proximate groups and issues [24–26]. Together, these expectations are highly consistent with extant studies of (non)violent extremist and terrorist groups, which together suggest that violence is positively associated with overall group size, broader organizational visibility, and more extensive network ties [7, 22, 23, 27]. We also note that these contentions closely parallel an emerging narrative on modern terrorism, which emphasizes the non-geographically constrained nature of terrorist groups and networks, as facilitated by “the accessibility of ideological information, training materials, finances, and radicalization assistance via modern transport and communications technologies” [13].

However, there is a strong literature which suggests the opposite, in that peaceful groups will be highly prone to communicate and mobilize over large distances [5, 28] whereas violent groups will be centered locally with very little engagement on the (inter)national scale [13, 29]. The logic to this alternate expectation is twofold. First, the government’s targeting of violent environmental groups for arrest and prosecution can often compel such groups to pursue decentralized leadership strategies so as to ensure that one group, cell, or member’s capture does not adversely affect the larger organization [30–32]. Second, the illegal and controversial tactics used by violent environmental groups likewise leave these groups wary of using formal mechanisms to communicate and/or coordinate across large distances, e.g. telephone or internet communications, which may be monitored by law enforcement, and to instead favor more informal and face-to-face forms of communication. While the latter mechanisms greatly help to facilitate trust between risk averse actors [28], they impose steep geographic constraints on intergroup interaction. As such, this literature suggests that violent environmental groups will be naturally decentralized and disinclined to engage in long distance communications. To test between these two hypotheses we will employ network data from a single UK radical environmentalist publication covering the years 1992 to 2003. The next section will describe the data employed.

## Data: Radical Environmental Groups in the UK

We focus on a single UK radical environmentalist publication, known as the “Do or Die” (DoD) magazine which was translated into machine readable format and has had formal networks extracted from the text by Almquist and Bagozzi [1]. Almquist and Bagozzi [1] chose to focus on this publication given its prominent place within the UK radical environmental movement during the 1990’s and 2000’s, as well as to ensure that their underlying documents, networks, and identified groups were as comparable as possible to one another. DoD was published semi-annually during the years 1992 to 2003 by an anonymous collective of radical British environmentalists based, in large part, in Brighton, United Kingdom (UK). At the time, the publication referred to itself as *the* voice of the UK Earth First! (EF!) movement. Later, its editorial collective suggested that the publication was rather only *a* voice of the movement [33]. As stated by an editor, DoD more generally “pushed a green anarchist, direct action perspective […] gave publicity to sabotage and had a no compromise attitude [and was] largely aimed at a few hundred people in the UK eco scene” although it was also widely read by more traditional anarchists and conservationists [33].

**Fig 1:**
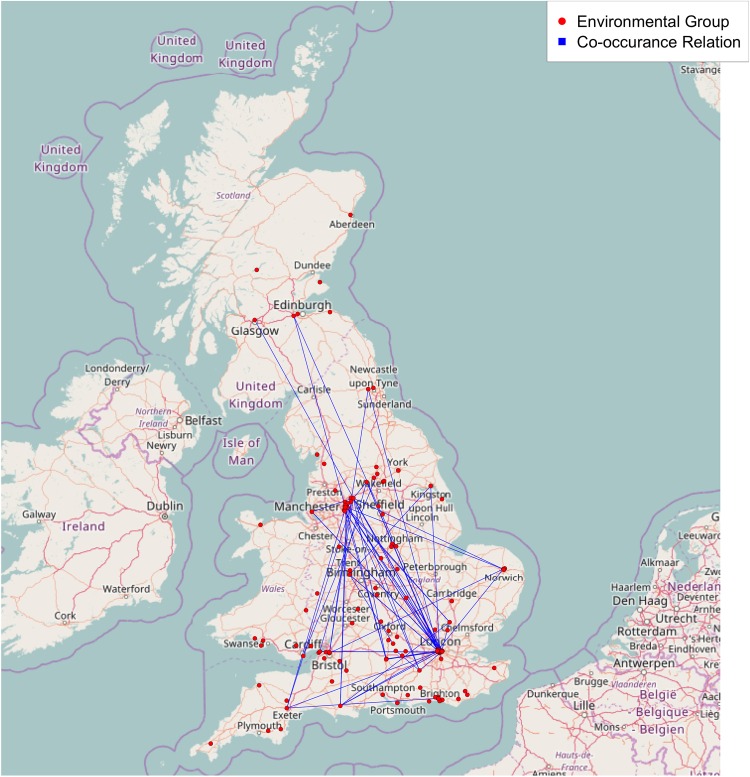
A map of the UK with the Radical environmental group network. Background map provided by the Open Streetmap project (http://openstreetmap.org).

Almquist and Bagozzi [1] define the network as a co-occurrence network consisting of 143 radical UK environmental groups identified in the text analysis portion of their article. The groups included in this network encompass a wide range of regional UK EF! organizations, anarchist collectives, anti-roads groups, and organizing venues. We fully list and describe each group included in this sample within the S1 Appendix. While some of these groups clearly speak to issues that fall outside of the environmental arena, we follow Almquist and Bagozzi [1] to refer to each group as a radical environmental group here, given their each being listed as a key contact, and frequently referenced in the text, within the DoD publication. We then supplement Almquist and Bagozzi’s [1] co-occurrence network with our own codings of each group’s geographic location within the UK, as determined by the group-specific mailing addresses listed in the contact information sections of DoD issues 6-10. The spatial layout of these connections can be viewed in Fig 1, where we find that our spatial network encompasses a diverse range of sites in England, Scotland and Wales, with major group concentrations in the greater London and Manchester areas.

## Methodology

In the following section we will detail our use of text analysis, network analysis, and spatial-network models in order to clarify and evaluate our competing hypotheses. We begin with a careful discussion of how we identify *violent* and *nonviolent* groups. We then follow up this subsection with a brief description of the geographic and topological characteristics of our network. This section is then followed up with a detailed account of our spatial network modeling approach and analysis.

### Violent versus Nonviolent Groups

As mentioned above, our methodological approach first seeks to objectively classify the groups in our spatial network as violent or nonviolent groups, for use within our spatial network analysis below. To classify groups in this manner, we use information from the DoD text corpus itself. This classification process requires that we identify passages in the 10 issue DoD corpus that can reasonably considered to encompass violent or nonviolent protest activities, and then evaluate the statistical association of each group with these violent and nonviolent passages. To do so, we apply unsupervised topic models so as to (i) uncover the latent themes or “topics” that are discussed across the DoD corpus and (ii) associate these topics with our UK environmental groups’ appearances in the text. Topic models allow one to recover the former quantity by treating one’s documents as combination of multiple overlapping topics, each with an associated set of words. Latent topics are then estimated via a hierarchical model that treats each document as a finite mixture of underlying topics; returning the words most strongly associated with each topic across all documents. We favor the recently developed STM [2] for these tasks. The STM estimates latent topics using the framework discussed above, while also incorporating document-level information via external covariates. For our application, the STM’s advantages intuitively lie in its ability to incorporate environmental group-based information—namely a document-indexed indicator of group occurrences—as binary predictors of attention towards different protest strategies and tactics across documents. This, in turn, allows us to estimate a set of topics across all documents, and to then evaluate whether the presence of a given group in a document significantly increases the attention dedicated to a given topic. When it does, we interpret this as evidence for a group being associated with the protest strategies that underlie that topic.

Our topic model classification approach thereby seeks to (i) uncover the latent violent protest activity topic(s) underlying the “Do or Die” (DoD) publication texts in an unsupervised fashion, (ii) identify the occurrence of UK environmental groups within these same texts, and (iii) measure the strength of association (and the degree of uncertainty about this association) of each group with the identified violent protest activity topic(s). We then classify a radical UK environmental group as using violent protest activities if we find that that group has a significant positive association with our identified violent protest activity topic(s). The first step in this methodological approach is to therefore convert all DoD publications to machine readable text for the unsupervised identification of latent topics within these texts. In this section of the supplementary material, we first provide an overview of the topic-model based classification approach that we use to determine whether (or not) the individual groups in our UK environmental group sample are associated with violent protest activities. This is followed by a presentation of the fifteen latent (text) topics that are obtained from this approach, as well as a more detailed discussion of the three topics that we identify as most indicative of violent protest activities. Lastly, we discuss how these three “violent topics” are used to classify each environmental group as a violent or nonviolent group, and provide the final list of UK environmental groups with their classifications. Please see the supplementary material for the full details. We next record whether (or not) a group was referenced within each individual page of our preprocessed texts. After doing so, we apply the STM to these inputs and find that, of the 143 environmental groups, only 19 are found to be violent using this method. This number, while small, is consistent with Gerstenfeld et al.’s [9] study of online extremist group websites, which likewise found that only a small share (16%) of such sites (and thus groups) exhibited violence. We list each of our identified violent and nonviolent actors in Table 1.

**Table 1:** List of violent and nonviolent environmental groups as designated by topic model classification, for full details see the supplementary material.

**Violent**				
cardiff ef!	reclaim the streets	glasgow ef!	the land is ours	haringey solidarity group
leeds ef!	reclaim the valleys	hunt saboteurs association	tyneside action for people and planet	no opencast
lune ef!	york ef!	london greenpeace	anarchist black cross	rising tide
manchester ef!	south somerset ef!	road alert!	campaign against the arms trade	
**Nonviolent**				
avon gorge ef!	arun valley ef!	uk subs	menwith womens peace camp	movement against the monarchy
bath ef!	bristol ef!	wolves eco action	undercurrents	newham monitoring project
beal valley rescue	cambourne ef!	cheltenham ef!	anarchist teapot action kitchen	1 in 12 club
blackburn ef!	chichester ef!	exeter environmental network	blatant incitement collective	opm support group
cambridge ef!	environmental ploughshares	hull on earth	disabled action network	partizans
east devon ef!	fife ef!	southampton ef!	solidarity federation	portsmouth anarchist network
faslane peace camp	genetic engineering network	woodland awareness and defence	urgent	primal seeds
forest action network	green anarchist network	irwell valley ef!	anarchist federation	radical routes
gwynedd and mon ef!	guildford ef!	norwich direct action forum	anarchist teapot mobile kitchen	sexual freedom coalition
hereford earth action	gwendraeth valley ef!	dartmoor ef!	autonomous centre of edinburgh	simon jones memorial campaign
leaf	head state support group	grampian ef!	brighton against benefit cuts	stop huntingdon animal cruelty
mid-somerset ef!	hereford ef!	london reclaim the streets	cage	tapol
newcastle ef!	hillfort ef!	newcastle tyneside action for people and planet	campaign to close campsfield	third battle of newbury
no m66	legal defence and monitoring group	norfolk and waveney ef!	class war	west london anarchists and radicals
norfolk ef!	liverpool ef!	sheffield ef!	chiapas link	worthing anarchist teapot
oldham ef!	making waves	swan network	english collective of prostitutes	anarchist youth network
oxford ef!	nottingham ef!	warwickshire action group	5th may group	the campaign to free vanunu
south downs ef!	parents action network brambles housing co-op	alf supporters group	56a infoshop	class_war_federation
the flat oak society	reading roadbusters	primitivist network	friends, families and travellers	direct action against the war
upper nene ef!	reclaim europe!	campaign against runway 2	friends of people close to nature	intercourse: talking sex
warwick ef!	save the hillgrove cats	the ecologist	i-contact video network	no platform anti-fascist network
wolves ef!	south devon ef!	industrial workers of the world	kate sharpley library	peat alert!
advisory service for squatters	stropp	justice?/schnews	kebele community centre	solidarity south pacific
anarcho-primitivist network	swansea people ef!	lamb	lancaster anarchist group	wild things
anti-fascist action	trident ploughshares 2000	mcspotlight	london animal action	cardigan bay ef!

### Topological and Geographic Characteristics of the Network

Social networks or graphs are both a way of theorizing about social structure, and a way of measuring and modeling the social world [34, 35]. A network is described in terms of two sets: (i) a vertex or node set (e.g., individuals or groups), and (ii) an edge set or relational set (e.g., friendship or communication). In the following section we briefly discuss the descriptive properties and metrics of interest (e.g., identifying the most active organization) for the network under consideration. First, we provide a basic set of descriptive statistics for the network. The co-occurance network for our environmental group sample is undirected with 143 nodes. It has a density (observed edges divided by number of possible edges) of *δ* = 0.006 and a mean degree (which is simply proportional to density) of $\bar{d}=0.86$. The largest component is of size 37 and a *δ*_*LC*_ = 0.09 [1].

Of key interest to social network analysts and to social scientists more generally is the question of who is in a position to broker between groups (also known as betweenness centrality) or who is in a position to access resources/contact the largest number of organizations? One way to frame this question for our application is in terms of network centrality, such as degree centrality (e.g., the individual with the most friends) or betweenness centrality (e.g., the individual with the strongest brokerage position.) [36]. Of significance here is the relationship between violent/nonviolent labeled groups and their centrality score. In Table 2 we report the top groups by degree and betweenness alongside the violent/nonviolent group label. Here we see that the most central actor in both betweenness and degree centrality is characterized as violent, and the remaining top ten most active groups are equally split between the violent and nonviolent labeled groups. To understand how this affects the interaction between groups we explore the level of direct homophily (not controlling for spacial interaction).

**Table 2:** Table of top 10 degree and top 10 betweenness groups, alongside each group’s violent/nonviolent designation within the radical environmental co-ocurrance network. In the label column: V designates violent and NV designates nonviolent.

Groups	Degree	Label	Groups	Between	Label
Reclaim The Streets	13	V	Reclaim The Streets	646	V
Class War	9	NV	Class War	536	NV
Norfolk Ef!	6	NV	No Opencast	256	V
Anarchist Black Cross	6	V	Leeds Ef!	202	V
The Land Is Ours	5	V	1 In 12 Club	198	NV
The Ecologist	5	NV	Avon Gorge Ef!	106	NV
Haringey Solidarity Group	5	V	The Ecologist	100	NV
Anarchist Federation	5	NV	The Land Is Ours	97	V
1 In 12 Club	5	NV	Anarchist Black Cross	96	V
Leeds Ef!	4	V	Tyneside Action…	87	V

While centrality measures (e.g., betweenness, closeness, and eigenvector) are used to measure the importance of a node’s position in the network, another metric of interest to network scientists is that of the clustering coefficient [37]. The clustering coefficient is not a centrality measure, since it does not measure the importance of a node’s position in the network but the extent to which one’s ties have ties with one another. Global clustering is the ratio of the triangles and connected triples in the graph, which for our network it is 0.294. This is low compared to small world networks as coined by Watts and Strogatz [37]. The average of the local clustering is 0.50 and can be interpreted as an indication that this network is not a so called small world network, since the clustering coefficient is statistically significantly smaller than one would expect given a random graph [37]. This evidence suggests that the violent and nonviolent groups in our sample may indeed exhibit distinct patterns of coordination, relative to the alternative of each group in our sample being linked by short chains of associated groups. However, to ascertain this more directly, we must examine our network’s properties conditional upon our violent/nonviolent group distinctions.

To implement the tasks outlined above, we compute a mixing matrix, which can be defined as the tabulation of within group ties in comparison to between group ties. With this metric in hand, we can test whether there is a high or low level of homophily for our network by performing a z-test on the mixing matrix and comparing the expected number of edges (under a random graph) to the observed number of edges. We find that that there are lower than expected (though not statistically significantly so) levels of mixing within group and higher than expected levels of between group mixing (again, not statistically significantly so). For further details see the S1 Appendix, section on Extended Descriptives of the Topological and Geographic Characteristics of the Network. We interpret this as suggesting that there are low levels of topological homophily in these groupings with a slight tendency towards heterophily. This provides a degree of preliminary evidence to suggest that the violent and nonviolent groups in our sample do not heavily weigh each other’s tendencies towards violent extremism when pursuing coordination strategies. However, the above metric of interaction does not control for spatial relations and thus cannot definitively determine whether the levels of homophily that we have identified are truly due to anti-mixing effects as opposed to, e.g., groups’ more general geographic proximities. To explore this we turn to parametric modeling of the network.

## Network Models

In the classic network literature —otherwise known as Social Network Analysis (SNA) or Network Science— networks have been considered largely in an “a-spatial” manner such that the geographical locations of network entities (and/or their interactions) has not been part of the analysis. This neglect has largely been due to lack of data and to the complication of managing and modeling such data. However the inclusion of spatial data within network analysis is rapidly changing as new software and GIS-type systems become available to general user.

Here, we consider a network as a mathematical object defined by a node set—also referred to as a vertex set—(e.g., individuals or organizations) and an edge set (e.g., friendship or collaboration). A network can be represented by a square binary adjacency matrix (*Y*), such that node *i* and node *j* have a relationship if *Y*_*ij*_ = 1, but do not have a relationship if *Y*_*ij*_ = 0. Typically the diagonal is treated as *NA* or undefined. In this work, a *spatially embedded* network is one whose nodes and/or edges are associated with geometrical objects (e.g., points, lines, or polygons) in a well-defined space. While geographical embedding spaces are of obvious interest, other sorts of spaces (including latent spaces, see for example [38], and attribute or “Blau” spaces [39, 40]) are also possible.

Geographically embedded networks occur in many social science applications, most notably within analyses of large-scale social systems. Geographic factors play fundamental roles in developing social structure in large-scale populations (e.g., [41–48]). In statistics, spatial structure has been (i) considered as a powerful covariate, (ii) used for predictive purposes, and (iii) included as a control when modeling social outcomes of interest (e.g., disease transmission). This is the basis for latent space models [38] for social networks, which associate nodes with points in a “latent” metric space where the probability of an edge between two nodes is a function (often logistic) of a metric distance between them with the idea that this distance can stand in for “latent” information between these two entities. When distances between actors are observed (e.g., physical space), they can likewise be employed as predictors/controls of network structure. We consider one such family, the *spatial Bernoulli graphs* [49], in the next section (for a more detailed discussion of these models, see the S1 Appendix).

### Spatial Network Models

Here, we focus on the *spatial Bernoulli graphs* introduced by [49] and used successfully in the network literature for prediction and inference in number of different contexts (e.g., [50–55]). For a more detailed discussion on these models, see the S1 Appendix. This method is one of the most direct ways of incorporating spatial effects into network modeling. Following the notation of Butts and others [53, 54, 56], we begin by positing a parametric function, $F_{d}(d,\psi )$, that (for some real parameter *ψ*) maps the distance between two nodes (*d*) into the probability of an *i*, *j* edge. This underlying function is referred to in the literature as the *spatial interaction function* (SIF) and its form governs the relationship between the spatial distribution of nodes and the network structure. This will be discussed in detail in Section. Spiro, Almquist and Butts [54] define the canonical parameters $\eta (\psi ,d)=\text{logit}F_{d}(d,\psi )$, where we may write the pmf for the random graph *G* with support G (represented via its adjacency matrix Y∈Y) as,

$$\begin{array}{c} \mathrm{Pr}(Y=y\;|\;D,\psi )=\prod_{\left\{i,j\right\}}B(y_{ij}\;|\;F_{d}\left(d_{ij},\psi \right)) \end{array}$$ (1)

with *B* as the Bernoulli pmf, $\psi \in R^{p}$ and *D* is a distance matrix on the elements of *V*. Such models are referred to as *spatial Bernoulli graphs*, and constitute the most basic family of spatial network models. This is the form we will use in this paper to recover the spatial properties of our network.

Spatial Bernoulli graphs can be thought of as nonlinear regression models with a conditionally independent dichotomous response and a link function corresponding to the SIF. Thus they are a special cases of *gravity models* [57], which model the expected interaction strength between nodes as a product of node potentials and an impedance function, i.e., $E\left[Y_{ij}\right]\propto P\left(i\right)P\left(j\right)F_{d}\left(D_{ij},\psi \right)$.

### Spatial interaction functions

In the previous section we noted that the SIF ($F_{d}$) is the heart of our modeling framework. It controls how distance relates to a marginal tie probability and its form strongly influences a network’s resulting structure [49]. Many different forms for the SIF are possible. Numerous empirical studies of various types of networks (e.g., [41–46, 58–60]) have shown that the marginal tie probability in social networks generally decreases as distance between individuals increases. As Butts and Acton [52] point out, “SIF selection is an inherently theoretical exercise: each functional form entails specific theorized relationships between distance and tie probability, along with their corresponding implications for network structure.” For example, consider a simple power law SIF,

$$\begin{array}{c} F_{d}\left(x,\left(p_{b},\alpha ,\gamma \right)\right)=\frac{p_{b}}{{\left(1+\alpha x\right)}^{\gamma }}, \end{array}$$ (2)

where 0 ≤ *p*_*b*_ ≤ 1 is a baseline tie probability, *α* ≥ 0 is a scaling parameter, and *γ* > 0 is the exponent which controls the distance effect. Butts and Acton [52] point out that the power law SIF is monotonically decreasing in distance which typically produces local clustering. For careful comparison of different SIFs considered in the literature, see [52].

## Analysis and Results

To fit the data to the above model and to adjudicate between potential SIFs in this work we will invoke the likelihood assumption for network models (similar to [61]), and use the model selection method of BIC [62] to find the best fitting model. We employ the algorithm and methods developed by Spiro, Almquist and Butts [54] to perform MLE for our spatial Bernoulli graphs with and without covariates. To perform our analysis we begin by modeling the complete network to see which SIF best explains the observed data. The results can be seen in Table 3, where we find that the *arctangent law* SIF is the best fitting model. This corresponds to the following probability function:

$$\begin{array}{c} \mathrm{Pr}\left(Y_{ij}=1\right)=F_{d}\left(x\right)=p_{b}(1-\frac{2}{\pi }\tan^{-1}\left(\alpha x\right)), \end{array}$$ (3)

where *p*_*bij*_ = logit^−1^(*θX*_*ij*_), *α*_*ij*_ = exp(*ψW*_*ij*_), *θ*, *ψ* are parameter vectors, and *X* and *W* are covariate matrices.

**Table 3:** BIC Selection table of all considered models.

Model Form	BIC
arctangent law	775.981
exponential decay law	776.039
power law	785.207
attenuated power law	785.278

**Fig 2:**
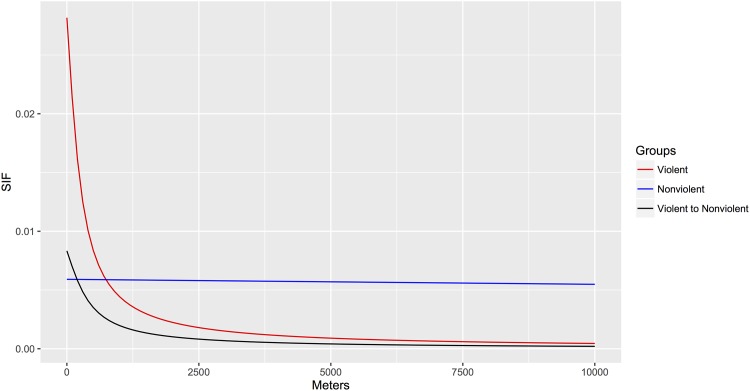
Probability plot for the SIF for the violent, nonviolent and interaction between the two groups.

We follow up with a confirmatory model of the *arctangent law*, where we distinguish between the violent and nonviolent organizational homophily and their cross tie interaction in both the scale parameter and the probability of interaction. The parameter estimates of this resulting model, SEs and P-values can be found in Table 4. Specifically, we find that nonviolent groups are almost a-spatial (i.e., have a very flat SIF that is only slightly decreasing over long distances), and that violent groups are highly spatially constrained, primarily interacting at shorter distances. Further, we find that the interaction between these two groups is highly structured by space at a rate that is lower than the internal interaction of either group by itself. While this is a very sparse network, violent organizations are also consistently more likely to connect with other violent organizations in the same city or neighborhood (i.e., less than a kilometer) than nonviolent organizations are to connect to other nonviolent organizations. This dynamic reverses as distance grows, wherein nonviolent groups increasingly become more likely to interact, relative to violent groups. Interaction between the two groups is quite low over even short distances. More concretely, at the neighborhood level (less than 600 meters) we can see that violent to violent group interaction probability is 0.007 versus 0.0058 for nonviolent group interaction. However, similar to above, this effect changes at distances of over a kilometer, where the probability of interaction is 0.0044 between violent groups and 0.00586 between nonviolent groups. We can observe these trends in Fig 2, where we see that nonviolent organizations have more interaction at almost all distances, and find a very steep-tail for the interaction between violent organizations, and violent and nonviolent organizations.

**Table 4:** Parameter table for the SIF for *arctangent law* functional form with homophily terms for violent to violent interaction, nonviolent to nonviolent interaction, and cross group interaction violent to nonviolent (and vice a versa due to the symmetry in the network).

	$\hat{\theta }$	SE	Pvalue
*p*_*b*_(*violent* ↔ *violent*)	-3.54	0.78	0.00*
*p*_*b*_(*nonviolent* ↔ *nonviolent*)	-5.13	0.11	0.00*
*p*_*b*_(*violent* ↔ *nonviolent*)	-4.78	0.35	0.00*
*α*(*violent* ↔ *violent*)	-5.53	1.64	0.00*
*α*(*nonviolent* ↔ *nonviolent*)	-11.39	11.18	0.31
*α*(*violent* ↔ *nonviolent*)	-5.97	0.93	0.00*

* signifies significance at the *p* < 0.05 level.

This difference in interaction has the potential to have large effects on the coordination, interaction, and diffusion of information for these types of organizations. These results confirm our second hypothesis, in demonstrating that violent actors are highly localized and likely decentralized in this context. As an extension, we evaluate the robustness of these findings to a number of alternate model specifications in our S1 Appendix. Notably, we find in these respects that our core results are robust to (i) a separate two-period analysis of our spatial network and (ii) a re-estimation of our primary model when controlling for overall levels of shared common interests among our groups (see the S1 Appendix for details). In the next section we will dissect our core findings, and their substantive implications.

## Summary and Discussion

Our analysis demonstrates that there is a spatially-controlled homophilous tendency for both the violent and nonviolent groups in our sample and that the corresponding network is structured by space. These conclusions, and the more specific results presented above, contribute to our current understandings of social movements, environmental politics, and (violent) extremist groups in a number of manners. First and foremost, our finding that violent and nonviolent environmental groups eschew coordination with one another—especially over moderate distances—is consistent with much of the conventional political-sociological thinking surrounding the UK environmental movement at this time, wherein more moderate environmental groups were often characterized as being wary of alliances with radical environmental organizations, primarily due to concerns over how such ties might affect the former’s reputation vis-à-vis the UK government [6]. More generally, the lack of cross-group coordination among violent and nonviolent groups that we identify is also consistent with theories of attribute-based segregation within social networks [3], as well as with anecdotal accounts of the tensions associated with cross-group collaboration when differing perspectives exist among environmental groups [4, 5].

Our finding that geographic constraints are substantially *more limiting* for *violent* environmental radical organizations contradicts several broader theoretical claims and empirical findings discussed within the social movements and terrorism studies literatures [7, 22, 23, 27], but is highly consistent with previous spatial analyses of radical environmental groups. For instance, the finding that the violent environmental groups in our UK group sample concentrate the vast majority of their coordination activities within a short distance is strikingly similar to the findings reported in Smith et al.’s [29] geospatial analysis of U.S.-oriented environmental terrorist groups. In this regard, Smith et al. notably find that over half of the environmental terrorists in their dataset and sample lived within 30 miles of their targets [29] and that 65% of these environmental terrorists’ antecedent activities also occurred within 30 miles of subsequently perpetrated incidents [29]. Hence, it would appear that violent environmental groups in the UK and environmental terrorists in the U.S. each exhibit comparably localized geographic patterns of activity and interaction. Similar spatial findings have been reported for the behaviors of international and Islamic terrorists as well [13, 29]. We take these similarities to suggest that, like U.S. environmental terrorists and related terrorist organizations, very localized patters of interaction exist among violent environmental radicals in the UK, and as such, their geographic reach is likely to be fairly limited.

There are several potential explanations for this specific finding. One possible explanation—suggested by both our UK-specific results and the broader literature [31, 32]—is that violent radical groups may avoid the use of mainstream communication technologies—such as the internet and personal telephones—during their coordination efforts, at least relative to the usage rates of these technologies by nonviolent groups. Indeed, a preference for face-to-face communication and an avoidance of unfamiliar contacts during collaboration are common tactics employed by criminal and terrorist organizations when such groups anticipate monitoring, infiltration, and disruption by law enforcement organizations [31, 63]. The fact that we find that our results hold in the S1 Appendix even after dividing our sample into pre and post-internet periods tentatively suggests that these divergent dynamics may remain even after the rise to prominence of internet communications among both violent and nonviolent groups. Given governments’ disproportionate targeting of *violent* environmental groups for arrest and prosecution, a second and related explanation offered by the literature suggests that the prospects of this form of scrutiny can more generally compel violent groups to disproportionately favor decentralized leadership strategies, so as to ensure that individual arrests and infiltrations do not adversely affect the broader organization [30–32]. Taken together, these explanations are thus consistent with a commonly noted (e.g., [64, 65]) tradeoff faced by dark networks in choosing between increased effectiveness (via the use of electronic communications) and avoiding detection (via the favoring of face-to-face communication over electronic mediums), and imply for our application that violent UK environmental organizations may favor the latter strategy when considering collaboration with like-minded groups.

The preceding discussion also suggests that our findings have several direct policy implications. Given the often illegal nature of violent protest tactics, we have noted above that governments frequently seek to disrupt or constrain the use of such tactics by radical groups [29, 66]. To the extent that our spatial findings concerning *geographically constrained* violent environmental group-collaboration also speak to the spatial location of violent environmental organizations themselves—and/or the locations of the actual violent actions that they perpetrate—the insights discussed above may aid governments in their efforts to eventually forecast, disrupt, and/or pre-empt these violent actions. Moreover, if violent radical groups do indeed adopt more spatially constrained collaboration networks in anticipation of government surveillance (as suggested above), our results also imply that law enforcement agencies may need to adapt their policies towards these groups over time, in light of this tendency for violent radical groups to alter their spatial collaboration behaviors in order to avoid detection and infiltration. This point notwithstanding, our core finding with respect to the highly spatially constrained nature of violent radical group coordination in our sample—relative to that of nonviolent groups—strongly suggests that sustained government monitoring and scrutiny may help to limit the geographic reach of violent environmental organizations.

Lastly, our finding that violent groups—and violent-to-nonviolent groups—are more likely to engage in coordination and communication if they are geographically proximate, but exhibit a quickly decreasing probability of interaction over even a short distances, is highly consistent with past findings concerning the spatial clustering of social movements and their spatial diffusion. This notably includes Gould’s [67] research into the effects of spatially confined Paris neighborhoods upon the emergence of violent insurgent networks, Hedstrom’s [68] similar identification of local neighborhood effects within the diffusion of Swedish trade unions, and McCadam’s [69] earlier research into the determinants of participation within high-risk activism. Excluding nonviolent groups’ interactions with one another, our findings in this regard are also at odds with the common characterization of local (radical and moderate) environmental groups as having a strong, and extensive, national-level ties (e.g., [5, 28]). That is, contra to the patterns suggested by existing environmental movements research, we find that violent radical environmental groups in our sample are very locally concentrated. This implies that, although such environmental groups often purport to “think global,” they continue in many respects to “act local.” We cautiously interpret this spatial finding as evidence in support our earlier contentions that our more systematic quantitative analysis of radical environmental groups can provide novel, and complimentary, insights to the existing qualitative research in this area.

Beyond these specific insights, the results discussed above also suggest several more general conclusions. While previous research into extremist groups has explored a number of group-level traits that may distinguish between violent and nonviolent groups (and actions) [7], our study is one of the first studies to explicitly compare and contrast the spatial network configurations of violent and nonviolent radical groups—environmental or otherwise. In this respect, we find spatial proximity to be a key factor in shaping radical environmental group networks—and especially violent environmental group networks. This finding, and its robustness to our two period analysis (as presented in our S1 Appendix), contradicts a long-standing narrative surrounding violent extremist groups—including violent left-wing extremists, pre-9/11 Islamic terrorists, and right-wing radicals—which largely characterizes such groups as spatially unconstrained and geographically decentralized [8–12, 70–72]. As such, our work follows more recent, sociospatial analyses of Islamic terrorist networks [13] and U.S. domestic extremist groups [29] in demonstrating that, for networks of violent radicals, local geographic constraints remain key.

This study also illuminates several specific avenues for future extension. The spatial findings discussed above notwithstanding, past research has also demonstrated that radical and moderate environmental groups in the UK vary in their cooperation with one another over time, owing largely to temporal variation in the perceived openness of political opportunity structures [6]. While we briefly examine this potential for temporal heterogeneity in our two period robustness analysis, subsequent efforts to further unpack patterns of violent and nonviolent environmental coordination over both space *and* time would likely provide a more insightful, and complete, picture of radical environmental group collaboration. Cross-national comparative studies of the spatial organizational structures of environmental groups have similarly revealed that these structures vary according to different (national) institutional environments [28]. It would thus be intriguing to evaluate the generality of our findings for radical environmental groups operating within a broader set of countries and institutional contexts, such as the U.S. Canada, and Germany. Finally, and in light of several intriguing spatial-network analyses of transnational terrorist networks [13, 73], a future evaluation of UK radical environmental groups’ *international* ties and spatial networks represents an especially promising area of anticipated research.

## Supporting Information

S1 Appendix For complete details on the UK environmental groups included in this sample, and on the methods used to classify violent and nonviolent groups, please see the S1 Appendix. (PDF)
