# Supplementary material for: The Spatial Properties of Radical Environmental Organizations in the UK: Do or Die!
Source: PLoS One. 2016 Nov 29;11(11):e0166609. doi: 10.1371/journal.pone.0166609 (PMC5127524; doi:10.1371/journal.pone.0166609)
Supplement: S1 Appendix — (PDF) [file pone.0166609.s001.pdf]

Supplemental Appendix for: The Spatial Properties of Radical Environmental Organizations in the UK

Zack W. Almquist[1,*], Benjamin E. Bagozzi[2]

1 Department of Sociology, School of Statistics and MPC, University of Minnesota, Twin Cities, MN, USA
2 Department of Political Science and International Relations, University of Delaware, Newark, DE, USA

Introduction

In the supplementary material provided below, we first present descriptions of the complete set of UK environmental groups that are included in our radical environmentalist group network. Note that while some of these groups clearly speak to issues that fall outside of the environmental arena, we follow Almquist and Bagozzi [1] to refer to each group as a radical environmental group here given their being listed as a key contact, and frequently referenced in the text, within the DoD (radical environmentalist) publication. We then provide an overview of the topic-model based classification approach that we use to determine whether (or not) the individual groups in our UK environmental group sample are associated with violent and/or illegal protest activities. This is followed by a presentation of the fifteen latent (text) topics that are obtained from this approach, as well as a more detailed discussion of the three topics that we identify as most indicative of violent and/or illegal protest activities. Next, we discuss how these three violent and/or illegal protest topics are used to classify each environmental group as a violent or nonviolent group, and provide the final list of UK environmental groups with their classifications. Following this discussion we carefully review inhomogeneous Bernoulli graph models for spatial network analysis and conclude with an in-depth overview of the empirical analysis employed in the main article.

UK Environmental Groups in Sample

This section describes the 143 UK environmental groups that are included in our sample. Where applicable, information on each group or organization's background is taken directly from that group's description within the DoD magazine and/or from Almquist and Bagozzi [1]. Specifically, the following UK environmental groups and organizations are included in UK group sample:

1. **1 in 12 Club** is a Bradford-based, anarchist-oriented social club, described by DoD as a "[l]ong-running autonomous social centre" [2] pp. 377.
2. **56@ Infoshop** is a Walworth-based social centre described as an "[a]utonomous radical infoshop with excellent anarchist archive and much more" [2] pp. 378.
3. **5th May Group** is a UK-based group of "Kurdish and Turkish anarchists in exile [who c]ampaign on local issues and compulsory military service" [3] pp. 228.
4. **Advisory Service for Squatters** is a UK-based organization focused on providing legal assistance for the homeless.
5. **ALF Supporters Group** is a UK-based group that seeks to provide material and symbolic support to animal rights activists, and members of the Animal Liberation Front (ALF), that have been imprisoned in

the UK; in addition to promoting a more general understanding of these activists' activities. The group operates independently to the ALF.

6. **Anarchist Black Cross** is the UK-based arm of an international activist group that seeks to provide material and symbolic support to political prisoners.
7. **Anarchist Federation** is a UK federation of "[a]narcho-communists [dedicated to] the abolition of capitalism and the state" [2] pp. 377 and publisher of both a regular newsletter and the *Organise!* magazine.
8. **Anarchist Teapot Action Kitchen** appears to be an earlier (or related) manifestation of the Anarchist Teapot Mobile Kitchen (see below). Not much information is available online, but this organization's main activities appear to be oriented around providing free food and drink at gatherings, concerts, and protest events involving various extremist groups. The Anarchist Teapot Action Kitchen was maintained as a separate group from the Anarchist Teapot Mobile Kitchen (described below) in our analysis given that the DoD texts seemed to imply that these groups were distinct, wherein stories typically referenced each group without giving mention to the other.
9. **Anarchist Teapot Mobile Kitchen** is a Brighton-based social centre known for "[c]heesy pop music with cheap, organic vegan food for action camps, gatherings and radical events" [2] pp. 377.
10. **Anarchist Youth Network** is a UK-based youth organization that is known as "[t]he only revolutionary youth network in the UK. Set up independently by young people, for young people, not as a recruiting ground for saddo lefties" [2] pp. 377.
11. **Anarcho-Primitivist Network** is a radical primitivism group that opposes modern civilization and seeks to promote these, and related radical ecological viewpoints, through information dissemination and other activities.
12. **Anti-Fascist Action** is a UK-based group focused on serving as a "[p]olitical and physical confrontation to the far right" [2] pp. 377, in addition to publishing the *Fighting Talk* magazine.
13. **Arun Valley EF!** is the autonomous UK EF! group associated with the Arun Valley regional area.
14. **Autonomous Centre of Edinburgh** is a social centre that "[d]raws together many campaigns for social and ecological issues into revolutionary struggle to overthrow capitalism" [3] pp. 228.
15. **Avon Gorge EF!** is the autonomous UK EF! group associated with the Avon Gorge regional area.
16. **Bath EF!** is the autonomous UK EF! group associated with the Bath regional area
17. **Beal Valley Rescue** is an Oldham-area group. Very little information about this group was available, but they may be oriented towards protesting the construction of the Beal Valley golf course.
18. **Blackburn EF!** is the autonomous UK EF! group associated with the Blackburn regional area.
19. **Blatant Incitement Collective** is a Manchester-based group that "[e]ncourages people to organize themselves ecologically and without hierarchy by sharing skills, knowledge and inspiration" [2] pp. 377.
20. **Brighton Against Benefit Cuts** is a Brighton-based organization whose focus is on the "[r]esistance to all attacks on benefits" [2] pp. 377, in addition to disseminating related information.
21. **Bristol EF!** is the autonomous UK EF! group associated with the Bristol regional area.
22. **CAGE**, based in Nottingham, is a "relatively new group/network resisting all manifestations of the prison state" [3] pp. 228.
23. **Cambourne EF!** is the autonomous UK EF! group associated with the Cambourne regional area.
24. **Cambridge EF!** is the autonomous UK EF! group associated with the Cambridge regional area.
25. **Campaign Against Runway 2** a network of ecological direct action groups that sought to prevent the expansion of the Manchester Airport.
26. **Campaign Against The Arms Trade** A UK-based nongovernmental organization (NGO) described by DoD as a "[b]road coalition of people seeking an end to the UK's role in the international arms trade" [2] pp. 377.
27. **Campaign to Close Campsfield** is an Oxford-based human rights group whose efforts are focused on "[r]egular demonstrations and other events to close Campsfield immigration detention centre" [2] pp. 377.
28. **Cardiff EF!** is the autonomous UK EF! group associated with the Cardiff regional area.
29. **Cardigan Bay EF!** is the autonomous UK EF! group associated with the Cardigan Bay regional area, primarily formed to oppose drilling by Chevron off the coast of Wales.
30. **Cheltenham EF!** is the autonomous UK EF! group associated with the Cheltenham regional area.

31. **Chiapas Link** a a Bristol-based group whose efforts are focused on the provision of “[e]xcellent information about, and radical support for, the Zapatista struggle in Mexico” [2] pp. 377.
32. **Chichester EF!** is the autonomous UK EF! group associated with the Chichester regional area.
33. **Class War** are a group of “[i]nfamous anarchists of the 80s” [3] pp. 228.
34. **Class War Federation** is a UK-based far-left political-anarchist group that “exists to promote class consciousness and working class control. Produces newspaper of the same name” [2] pp. 377. Note that this group was referred to as “Class War” in pre-2003 issues of DoD. Class War Federation was kept as separate group entries here given the potentially changing nature of the underlying group.
35. **Dartmoor EF!** is the autonomous UK EF! group associated with the Dartmoor regional area.
36. **Direct Action against the War** is a UK-based “[e]mail list for exchanging information about direct action resistance to the war” [2] pp. 377.
37. **Disabled Action Network** is a UK-based group focused on the provision of “[d]irect action by and for disabled people” [2] pp. 377-378.
38. **East Devon EF!** is the autonomous UK EF! group associated with the East Devon regional area.
39. **English Collective of Prostitutes** is a UK-based “network of women working at various levels in the sex industry” [2] pp. 378.
40. **Environmental Ploughshares** is an Oxford-based ecological nonviolent direct action group.
41. **Exeter Environmental Network** is a UK ecological direct action group.
42. **Faslane Peace Camp** is a permeant peace camp situated in Argyll and Bute, Scotland, “[r]ight across the road from the nuclear sub base [whose members] stop convoys and generally make the military’s life awkward” [2] pp. 378.
43. **Fife EF!** is the autonomous UK EF! group associated with the Fife regional area.
44. **Forest Action Network** is a Norwhich-based environmental group focused on forest-issues.
45. **Friends of People Close to Nature** is a Herrtfordshire-based “[i]ndependent group working to support the struggles of indigenous peoples against development” [2] pp. 378.
46. **Friends, Families and Travellers** is a Brighton-based group known for “[w]orking towards a society where travellers can live on the road without fear of prosecution or harassment” [2] pp. 378.
47. **Genetic Engineering Network** is a network of UK-based activists known for the provision “[i]nformation for action, updated details of test site locations and support for local groups” [2] pp. 378 focused on opposing genetic engineering.
48. **Glasgow EF!** is the autonomous UK EF! group associated with the Glasgow regional area.
49. **Grampian EF!** is the autonomous UK EF! group associated with the Grampian regional area.
50. **Green Anarchist Network** is an Oxford-based ecological anarchist movement promoting local autonomy, with efforts directed against industry and pollution, amongst other activities.
51. **Guildford EF!** is the autonomous UK EF! group associated with the Guildford regional area.
52. **Gwendraeth Valley EF!** is the autonomous UK EF! group associated with the Gwendraeth Valley regional area.
53. **Gwynedd and Mon EF!** is the autonomous UK EF! group associated with the Gwynedd and Mon regional area.
54. **Haringey Solidarity Group** is a well established London-based radical organization of community activists, and publisher of the *Haringey Community Action* newsletter.
55. **Head State Support Group** is a UK-based support group focused on providing support and assistance to radical, ecological, and related activists who have been sectioned under the Mental Health Act in response to their protest activities.
56. **Hereford Earth Action** is a UK ecological direct action group.
57. **Hereford EF!** is the autonomous UK EF! group associated with the Hereford regional area.
58. **Hillfort EF!** is the autonomous UK EF! group associated with the Hillfort regional area.
59. **Hull on Earth** is a UK ecological direct action group.
60. **Hunt Saboteurs Association** is a “nationwide network of groups using direct action to stop fox hunting” [2] pp. 378, and publisher of the *Howl* magazine.
61. **I-Contact Video Network** is a Bristol-based group focused on facilitating “[a]utonomous and independent video production by activists” [2] pp. 378.

62. **Industrial Workers of the World** refers to the UK-based arm of the Industrial Works of the World (IWW), a radical labor union whose UK club is described by DoD as a "[r]evolutionary union whose aim is to gain control of workplaces and eliminate the bosses" [2] pp. 378.
63. **Intercourse: Talking Sex** is an Edinburgh-based "non-hierarchical organization devoted to encouraging people to develop comfortable and positive ways of thinking and taking about sex and sexuality" [2] pp. 378.
64. **Irwell Valley EF!** is the autonomous UK EF! group associated with the Irwell Valley regional area.
65. **Justice?/Schnews** is a Brighton-based weekly publication (Schnews) and related group (Justice) that reports on anarchist, environmental and social issues. Though we refrain from including publications within our master list of groups, *Justice?/Schnews* was included within DoD's more general UK contact-group list in some issues (likely owing to the Justice-group that prints Schnews), and is hence included here. We do however limit our co-occurance search queries to "Justice?/Schnews," as opposed to individually including an entry and reference to the publication "Schnews" in our co-occurence and group lists.
66. **Kate Sharpley Library** is a UK-based library, known for having "[t]he most extensive collection of anarchist material in the UK" [2] pp. 378.
67. **Kebele Community Centre** is a Bristol-based radical social centre.
68. **LAMB** is a Manchester-based UK group/network-contact listed by DoD.
69. **Lancaster Anarchist Group** is a Lancaster-based anarchist organization that is known to be "active in many struggles" [2] pp. 378.
70. **LEAF** is a UK ecological direction action group.
71. **Leeds EF!** is the autonomous UK EF! group associated with the Leeds regional area.
72. **Legal Defence and Monitoring Group** seeks to provide legal backup to UK (primarily London) based demonstrations, by monitoring police activities during protests and providing support to those arrested and/or facing trial for protest activities.
73. **Liverpool EF!** is the autonomous UK EF! group associated with the Liverpool regional area.
74. **London Animal Action** is a London-based group whose focus is on animal rights and the broader networking of, and information dissemination for, UK animal rights groups.
75. **London GreenPeace** is an environmental anarchist collective known for its anti-war, anti-nuclear, and anti-McDonald's protest efforts.
76. **London Reclaim the Streets** is the London-based chapter of this UK ecological direct action group, which is primarily oriented around protesting automobiles and globalization.
77. **Lune EF!** is the autonomous UK EF! group associated with the Lune regional area.
78. **Making Waves** is a Sheffield-based UK-group/network that was listed as a contact in some issues of DoD.
79. **Manchester EF!** is the autonomous UK EF! group associated with the Manchester regional area.
80. **McSpotlight** arose out of the British McLibel Trial and is a UK-based group associated with the international Anti-McDonald activist network of the same name.
81. **Menwith Womens Peace Camp** is a women-only direct action group, and associated protest-camp, that was established to promote peace and to protest the siting of cruise missiles at the US military base in Newbury.
82. **Mid-Somerset EF!** is the autonomous UK EF! group associated with the Mid-Somerset regional area.
83. **Movement Against the Monarchy** is a UK-based anarchist organization described by DoD as "[r]oyal hating class struggle anarchist ruffians" [2] pp. 378.
84. **Newcastle EF!** is the autonomous UK EF! group associated with the Newcastle regional area.
85. **Newham Monitoring Project** is a UK-based "[c]ommunity group giving support, advice and campaigning on issues of racial harassment and civil rights" [2] pp. 378.
86. **No M66** is a group-led anti-roads campaign in North East Manchester.
87. **No Opencast** is a UK-based anti-mining group known for "[c]ampaigning against opencast mining and networking information between similar groups" [2] pp. 378.
88. **No Platform Anti-Fascist Network** is a Leeds-based "[n]etwork of anti-fascist socialists, anarchists and anti-capitalists united by the policy of 'no platform for fascists"' [2] pp. 378.
89. **Norfolk and Waveney EF!** is the autonomous UK EF! group associated with the Norfolk and Waveney regional area.

90. **Norfolk EF!** is the autonomous UK EF! group associated with the Norfolk regional area.
91. **Norwich Direct Action Forum** is a UK ecological direct action group.
92. **Nottingham EF!** is the autonomous UK EF! group associated with the Nottingham regional area.
93. **Oldham EF!** is the autonomous UK EF! group associated with the Oldham regional area.
94. **OPM Support Group** is a UK-based group promoting "[p]ractical solidarity with the indigenous people of West Papua" and producer of an occasional newsletter detailing these efforts [3] pp. 229.
95. **Oxford EF!** is the autonomous UK EF! group associated with the Oxford regional area.
96. **Parents Action Network Brambles Housing Co-op** is a Sheffield-based support group.
97. **PaRTiZans** is a UK-based anti-mining group known for fighting "against the mining activities of the corporation RTZ [and a g]ood information resource" [2] pp. 378.
98. **Peat Alert!** is a group of Leeds-based environmental activists known for their "[e]xcellent and pretty successful campaign to halt peat extraction in the north England" [2] pp. 378.
99. **Portsmouth Anarchist Network** is a UK-based anarchist group known for discussing and organizing "support for prisoners, anti-militarism and workers in struggle" [2] pp. 378.
100. **Primal Seeds** is a Manchester-based group of anti-biotech activists, known for being "[a]ctively engaged in protecting biodiversity and creating local food security " [2] pp. 378.
101. **Primitivist Network** is a short-run UK primitivist group aligned with the Green Anarchist Network, and also publisher of the *Missing Link* journal.
102. **Radical Routes** is a Leeds-based leftist organization and network of housing co-ops. Known as a provider of information on government-sponsored housing.
103. **Reading Roadbusters** is a UK ecological direct action group.
104. **Reclaim Europe!** is a London-based group focused on protesting globalization and UK-Europe politics.
105. **Reclaim the Streets** is a UK ecological direct action group, primarily oriented around protesting automobiles and globalization.
106. **Reclaim the Valleys** is a South Wales-based UK ecological direct action group, known primarily for protesting open-cast mining and environmental destruction within the South Wales region.
107. **Rising Tide** Is a UK-based "network of independent groups and individuals taking local action, and building an international movement, against climate change" [2] pp. 378.
108. **Road Alert!** is a UK ecological support group, based in Newbury.
109. **Save the Hillgrove Cats** is a UK animal rights group whose efforts were focused on protesting and opposing the Oxfordshire-based Hill Grove Farm, a commercial breeder of laboratory cats.
110. **Sexual Freedom Coalition** is a UK-based leftist group that actively "[c]ampaigns against laws restricting all adult consensual activity" [2] pp. 378.
111. **Sheffield EF!** is the autonomous UK EF! group associated with the Sheffield regional area.
112. **Simon Jones Memorial Campaign** is a social justice campaign centered around the death of Simon Jones (a casual worker that was killed on his first day of work) that also seeks to protest the UK casual labour economy more generally.
113. **Solidarity Federation** is a Manchester-based leftist group, described by DoD as a "[a]mall network of anarcho-syndicalists" [2] pp. 379.
114. **Solidarity South Pacific** is a Brighton-based leftist organization known for promoting "[s]olidarity activity for tribal peoples and the ecology of the Pacific Rim" [2] pp. 379.
115. **South Devon EF!** is the autonomous UK EF! group associated with the South Devon regional area.
116. **South Downs EF!** is the autonomous UK EF! group associated with the South Downs regional area.
117. **South Somerset EF!** is the autonomous UK EF! group associated with the South Somerset regional area.
118. **Southampton EF!** is the autonomous UK EF! group associated with the Southampton regional area.
119. **Stop Huntingdon Animal Cruelty** is a UK-based animal rights group whose aim is "to close down Huntingdon Life Sciences, Europe's biggest animal testing laboratory" [2] pp. 379.
120. **STROPP**, also known as Stop The Road On Peaks Parnham, is a network of groups formed to oppose the Peaks Parkway project through Grimsby.
121. **SWAN Network** is the UK arm of the Social Work Action Network (SWAN).

122. **Swansea People EF!** is the autonomous UK EF! group associated with the Swansea regional area.
123. **TAPOL** is a UK-based human rights NGO whose focus is on publicizing human rights in Indonesia and East Timor.
124. **The Campaign to Free Vanunu** is a UK-based organization focused on supporting and freeing Mordechai Vanunu, an anti-nuclear whistle blower who was imprisoned by Israel after leaking details of the Israeli nuclear program to the British press.
125. **The Ecologist** is a more mainstream UK environmental journal. Though we refrain from including publications within our master list of groups, *The Ecologist* was included within DoD's more general UK contact list in some issues, and is hence included here.
126. **The Flat Oak Society** is a network of ecological activists that oppose the A299 Thanet Way bypass in Kent, among other activities.
127. **The Land is Ours** is a UK direct action land-rights group that advocates access to the land, its resources, and the planning process.
128. **Third Battle of Newbury** is a Newbury-based environmental and anti-roads group focused on "[c]ontinued resistance to road construction" [2] pp. 379
129. **Trident Ploughshares 2000** is an activist anti-nuclear weapons group, specifically focused on disarming the UK Trident Nuclear Weapons system.
130. **Tyneside Action for People and Planet** a Newcastle-based UK ecological direct action group, often self described as an awareness-raising nonviolent direct action group.
131. **UK Subs** is, to the best of our knowledge, an English Punk Band. Nevertheless, the group is listed under the general UK group contact list for DoD (Issue 7), as well as under similar contact lists for Earth First! action updates, and is hence included here.
132. **Undercurrents** is a former leftist video magazine producer, and later online-video/pirate-TV operator, located in Oxford.
133. **Upper Nene EF!** is the autonomous UK EF! group associated with the Upper Nene regional area.
134. **URGENT** is also known as the green field housing network, and is a UK support group.
135. **Warwick EF!** is the autonomous UK EF! group associated with the Warwick regional area.
136. **Warwickshire Action Group** is UK ecological direct action group.
137. **West London Anarchists and Radicals** is a "[l]ocal class struggle anarchist/communist group" [3] pp. 229 that also publishes a bi-monthly newsletter.
138. **Wild Things** is a Nottingham-based initiative, described by DoD as an "[e]xcellent and radical eco-education project" [2] pp. 379.
139. **Wolves Eco Action** is a UK ecological direct action group.
140. **Wolves EF!** is a UK ecological direct action group.
141. **Woodland Awareness and Defence**, also known as WAND, is a Dunbar-based UK ecological direct action group.
142. **Worthing Anarchist Teapot** is a Worthing-based social centre known for providing "free tea and coffee plus radical literature from squats and town centre stalls" [2] pp. 379.
143. **York EF!** is the autonomous UK EF! group associated with the York regional area.

Classification Approach & Input Quantities

As stated in the main paper, our topic model classification approach seeks to (i) uncover the latent violent[1] protest activity topic(s) underlying the "Do or Die" (DoD) publication texts in an unsupervised fashion, (ii) identify the occurrence of UK environmental groups within these same texts, and (iii) measure the strength of association (and the degree of uncertainty about this association) of each group with the identified violent protest activity topic(s). We then classify a UK environmental group as using violent protest activities if we find that that group has a significant positive association with our identified violent protest activity topic(s). The first step in this methodological approach is to therefore convert all DoD publications to machine readable text for the unsupervised identification of latent topics within these texts.

[1]Note that, in this analysis, we consider property destruction to be a form of violence.

We acquired the complete 10-issue corpus of the DoD publication from Almquist and Bagozzi [1]. Issues 5-10 and 1 were obtained by Almquist and Bagozzi [1] in machine readable form from the online archive "Eco-action.org", which was a website used primarily for documenting UK environmental environmental activities during the 1990's [4]. The entries are stored in HTML at the story-level (separately for each issue), and all individual stories were webscraped for use in the analysis below. PDF-images of the four remaining issues (Issues 2-4) were then obtained by Almquist and Bagozzi [1] from a second online archive of environmental extremist activities known as the "Talon Conspiracy" [5]. These PDF-image files were then converted to machine readable text using optical character recognition (OCR) software. Following Almquist and Bagozzi [1], we utilize the webscraped text for issues 5-10 and 1 in our analysis below, and then employ the OCR'd versions for the remaining issues (2-4).

Using these machine readable texts, our classification approach requires that we next create two intermediate input quantities from the text files described above: (i) a list of relevant UK environmental groups and (ii) a corpus of fully preprocessed text "documents." The former quantity is used for identifying the UK environmental groups that occur within our text corpus, and was obtained from the contact information listed at the end of each DoD issue (as described in the main paper)—yielding a total of 143 UK environmental groups. The latter quantity mentioned above is used for identifying the latent strategies that are discussed across our DoD corpus, including (potentially) violent protest activities.

Regarding the creation of our corpus of fully processed "documents," are decisions here closely follow the decisions and methodologies discussed in [1]. In these respects, we must first define what a standard document should be for the ensuing unsupervised topic model analysis. Based upon our own substantive knowledge of the DoD corpus—as well as the work presented in Almquist and Bagozzi [1] and similar applications of topic models to social science texts—plausible document designations include each individual DoD issue, each individual page of text within our DoD sample, individual sentences or paragraphs, or each individual story-entry within the corpus. Extant social science research has applied similar topic models to those used below to documents ranging in size from individual tweets [6] to individual books [7]. Others have used more arbitrary text-breaks to define documents such as page-breaks, sentences, multi-sentence sequences, or paragraphs [8–10]. Hence, for the methods applied below, a great deal of flexibility can be afforded in defining one's documents.

However, because topic models are inherently designed to analyze *large collections of text* [11], the limited number of actual DoD issues (i.e., 10) prevents us from treating the individual DoD issues as our documents of interest. This limitation, and the broader formatting idiosyncracies of DoD, accordingly made the designation of (comparable) documents for this corpus uncharacteristically difficult. To this point, recall that the individual contributions within DoD were often inherently unstructured and variable, with some entries following traditional paragraph form, others being more experimental and artistic in format, and still others following a simple bullet point style. Moreover, the stories and pages within DoD often have text blocks arranged in different paragraph and column formats, with individual story entries ranging from 21,000+ words to 140 words (or less). Complicating this further was the fact that the OCR'd documents in our sample (issues 2-4), which were OCR'd at the page-level, often lost information on paragraph breaks and story breaks whereas our webscraped documents (issues 1, 5-10) were archived at the story level, without complete information on page-breaks.

In light of these inconsistencies, and following Almquist and Bagozzi [1], the most defensible method for dividing the DoD corpus into text documents of comparable length is the use of multi-sentence sequences. Individual sentence-length documents were deemed far too short for identifying group occurrences or underlying mixtures of

topics. By contrast, the use of consecutive sequences of sentences has past justification in the estimation of underlying topics from text (e.g., [9,12]). In essence, this approach entails that one link together non-overlapping series of n consecutive sentences, treating each resultant sentence sequence as an individual document. As such, we started from the first DoD issue, and iterated through all 10 issues (in order), combining consecutive sentences by our designated n, and incrementally treating each subsequent n sentence sequence as a new document. While somewhat arbitrary, this process helps to guarantee that each document is of comparable size and content, which would not be the case if we were to have used story level breaks or page-level breaks [1].

While constructing these sentence sequence documents, we follow Almquist and Bagozzi [1] and remove the aforementioned UK (and international) environmental group contact lists from the final pages of each DoD issue. While we use these contacts for identifying our groups of interest, we wish to avoid treating this text as actual content text during either the extraction of group co-occurrence information or the topic modeling stages of our analysis, given that the contact lists included within DoD provide little to no surrounding content text aside from the listed names and contact information for each group. After dividing our remaining corpus into 12 sentence documents, we then processed each document's text to remove all punctuation, numbers, and stopwords. The removal of these character-sets is standard preprocessing for our intended topic model techniques [1,13–15]. In line with extant research [16–18], we then removed all sparse terms that do not occur in at least 1% of the documents in our corpus, converted all remaining words to lower case, stemmed our remaining words, and re-structured our corpus into a document-term-matrix. Taken together, these preprocessing steps created a corpus with 3,210 unique documents and 2,082 unique word-stems.

Our topic model analysis, and past research [1], ultimately informed our decision to use 12 sentence sequences as our primary document length. For this analysis, the choice of 12 sentence sequences yielded a document length that was comparable to the shorter, and most common, stories within the DoD corpus, while also minimizing the aforementioned heterogeneity in story-length documents. The choice of 12 sentence sequences also allowed us to maximize the number, and validity, of our environmental group occurrence indicator, which serves as the primary input for our group classifications. This occurrence indicator reports whether or not a group from our primary group list was mentioned within a given document in our corpus. As such, the length of a document directly affects our identified occurrences, and we believe 12 sentence sequences to be a reasonable balance between the identification of true occurrences and the avoidance of false occurrences. Indeed, lowering this sentence sequence threshold by 50%, to six sentence sequences, significantly reduces the number of identified occurrences, potentially ensuring that a substantial number of missed associations between occurrences and identified strategies. Increasing sentence-length by 50%, to 18, instead raises the potential for false positives, as UK environmental groups that are mentioned in one section of the original DoD text will be considered as having an association with a protest strategy they may in fact have been discussed in an entirely different context.

To construct our occurrence indicator, we then iterate through every group included within our UK environmental group list. For each group (i), we query each 12 sentence document (d) to record whether (=1) or not (=0) group i occurred in document d. Importantly for our documents d, we use the *unprocessed* 12 sentence sequence documents, as opposed to the fully preprocessed documents discussed above, as de-capitalization, stemming, and proper noun removal each undermine the appearance and identification of our target group names within our co-occurrence queries.[2] From

[2]We do, however, continue to omit the actual contact lists included at the end of each DoD issue

the results of these queries, we construct a binary i by d matrix, where 1's denote occurrences of groups (rows) in particular documents (columns), and 0's denote the absence of a group within a given document. These binary indicators serve as our group-specific variables in the analysis below.[3]

Topic Model Application

We next extract the underlying protest tactics that are discussed across the DoD corpus, and variation therein. To do so, we apply unsupervised topic models to our preprocessed text-documents so as to simultaneously (i) uncover the latent themes or "topics" that are discussed across documents and (ii) associate these topics with the UK environmental group occurrence indicator discussed above. Topic models allow one to recover the former quantity by treating one's documents as combination of multiple overlapping topics, each with a representative set of words. Generally speaking, latent topics are then estimated via a hierarchical model that treats each document as a mixture of underlying topics; returning the words most strongly associated with each topic across all documents.

We favor the structural topic model (STM) [13] for our present application. The STM estimates latent topics using the hierarchical framework discussed above, while also incorporating document-level information via external covariates into one's prior distributions for document-topics. As such, one can use the STM to not only identify a set of shared latent topics across a corpus, but also to evaluate potential relationships between document-level covariates and the prevalence of one's topics within and across documents. Accordingly, the STM has been effectively used to estimate the effects of survey-respondent characteristics upon variation in respondents' open ended responses [13], as well as in identifying the underlying themes of the same DoD corpus that is currently under analysis [1]. For our application, the STM's advantages relate to its ability to incorporate group-based information—namely our document-indexed indicator of group occurrences—as binary predictors of attention towards different protest strategies and tactics across documents. This, in turn, allows us to estimate a set of topics across all documents, and to then evaluate whether the presence of a given group in a document significantly increases the attention dedicated to a given topic. When it does, we interpret this as evidence for a group being associated with the protest strategies that underlie that topic.

We specifically model our 12 sentence-sequence document-corpus as a function of the aforementioned external group occurrence covariate using the `stm` R package [19]. As is the case for most unsupervised topic models, we must explicitly choose the number of topics to be estimated within the STM. Robert et. al. [19] note that "[t]here is no right answer to the appropriate number of topics. [...] For small corpora (a few hundred to a few thousand) 5-20 topics is a good place to start." As our corpus contains 3,210 documents, we rely on the above suggestions—as well as on the topics identified in earlier analyses of our texts [1]—to select a topic number of 15 for our primary STM analysis. To address multi-modality concerns, we follow Robert et. al. [13] and estimate a series of 50 separate 15-topic STMs using different starting parameters for each, and store the exclusivity and semantic coherence of topic words for each model.[4] We then choose a single model from this set of 50 models that maximizes the semantic coherence

from the construction of our 12 sentence sequence documents for this step, as including this text within our document sample for co-occurrence identification would lead to a large number of false positives.

[3] Note that we drop group indicator-variables for those groups that did not occur within any document.

[4] Exclusivity measures how exclusive one's topwords are to each topic based upon a word's relative probabilities of association across topics, whereas semantic coherence quantifies the relative co-occurrence of our topics' identified topwords across our corpus, and thus provides a sense of how internally consistent a topic is [13].

and exclusivity of our corresponding topic word vectors. Based on the results of this model, we next examine the sets of 20 topwords that best characterize each identified latent topic. After establishing the semantic meaning of our 15 identified latent topics according to these topwords, we derive a variety of post-estimation quantities that allow us to classify our UK environmental groups according to their use of violent protest strategies.

Identified Topics

Based upon the methodology and approach discussed above, our primary STM model will identify the 15 topics that best characterize our preprocessed 12 sentence sequence documents across the entire DoD corpus. Each topic represents an underlying word distribution wherein each word in our corpus is given a posterior probability of assignment to that topic. For these word vectors, and consistent with Robert et. al. [13], we extract the words most associated with each topic according to frequency exclusivity scoring metrics. We then substantively interpret the meaning of each topic based upon these probabilistic "top" word assignments. Specifically, we draw upon the twenty most frequent and exclusive words for each topic, and present these (stemmed) topword vectors for each of our 15 topics of interest—alongside our labels for each topic and the topic's reference number—in Fig 1.

Topic	Top 20 Words	Labels
1	one, made, anoth, everi, side, time, hand, mani, turn, left, open, togeth, enough, around, given, reach, great, strong, run, join	Inspirational Language
2	role, polit, organis, ideolog, non, activ, idea, movement, radic, mainstream, question, organi, opinion, individu, media, violent, problem, engag, potenti, posit	Group Identity Debates
3	oil, compani, mine, crop, farmer, fish, indigen, papua, corpor, genet, govern, zapatista, shell, bougainvill, peasant, mexico, engin, industri, western, indonesian	Neocolonialism
4	book, isbn, publish, box, http, zine, magazin, guid, press, copi, write, articl, send, email, read, page, list, web, review, mail	Eco−Literature
5	women, law, case, footbal, men, legal, game, evid, court, school, terrorist, privat, famili, properti, terror, intellig, record, team, agenc, custom	News and Culture
6	speci, habitat, restor, wildlif, peat, bird, ecosystem, biodivers, plant, soil, extinct, woodland, highland, conserv, hotspot, moor, natur, garden, biolog, wild	Species Conservation
7	let, away, will, back, voic, danger, keep, put, take, look, etc, earth, thank, mother, first, ever, fill, eye, die, sound	General Concern
8	forest, protect, log, water, environment, mountain, dam, north, nativ, area, east, island, region, rainforest, timber, ago, urban, northern, land, river	Land Conservation
9	polic, arrest, cop, crowd, march, banner, vehicl, window, offic, smash, demo, confer, riot, hour, demonstr, mask, bank, pragu, car, camera	Violent Protest
10	action, direct, campaign, group, involv, network, sabotag, anti, reclaim, tactic, meet, success, event, sab, act, target, rts, opencast, media, aim	Direct Action/Ecotage
11	camp, evict, site, road, quarri, twyford, tunnel, sit, council, tree, climb, squat, set, hill, fenc, hous, build, tarmac, built, trash	Occupation/Camps
12	cultur, must, societi, exist, life, learn, live, desir, civilis, understand, human, relationship, skill, can, experi, planet, process, sens, domin, alien	Sustainable Societies
13	pirat, prison, sentenc, black, panther, bomb, murder, jail, ship, imprison, kill, shepherd, sent, africa, gun, white, death, frame, fbi, trial	International Terror
14	know, dont, realli, want, thing, that, think, lot, someth, get, someon, sure, there, say, just, bad, theyr, didnt, ask, thought	Admonishments
15	capit, capitalist, labour, revolut, class, struggl, global, economi, union, counter, worker, social, econom, elit, resist, globalis, autonomi, wage, spanish, market	Anti−Capatalist Left

Fig 1. Topwords for 15-Topic Model.

By and large, the 15 topics identified by the STM appear to correspond to meaningful constructs that are each highly similar to those discussed in Almquist and Bagozzi [1]. For our application, we group the 15 identified topics into three general categories for discussion: "ideology", "tactics", and "general discourse." Ideology represents the largest of these topic groupings, and includes topics 2-4, 6, 8, 12, and 15. In line with our earlier discussions of the overarching goals of the DoD publication [20], Topic 2 is labeled **Group Identity Debates** and exhibits topwords pertaining to the ever-changing environmental perspectives and overarching goals of the UK EF! movement, and debates therein. Topic 4 is labeled **Eco Literature**, and encompasses topwords pertaining to the *publication and dissemination* of environmentalist viewpoints. The remaining ideology-based topics clearly identify ideological issues that environmentalists and radical leftists are either for (Topic 6: **Species Conservation**; Topic 8: **Land Conservation**; Topic 12: **Sustainable Societies**) or against (Topic 3:

Neo-Colonialism; Topic 15: **Anti-Capitalism**). We take the identification of these highly plausible ideological topics as evidence to suggest that our STM is performing as expected for the corpus as hand.

The next grouping of topics are of most interest to our group classifications, and encompass a number of protest tactics and strategies. In full, we identify four topics in Fig 1 that appear to pertain to protest tactics: Topic 9: **Violent Protest**; Topic 10: **Direct Action/Ecotage**; Topic 11: **Occupation/Camps**; and Topic 13: **International Terror**. The **Violent Protest** topic encompasses topwords related to mass protest ("crowd", "march", "demonstr", "banner") as well as to the potential violent consequences of such activities ("polic", "arrest", "cop", "smash", "riot"). **Direct Action/Ecotage** contains both "direct" and "action" in its topwords, in addition to words related to illegal and potentially violent activities such as "sabitag", "act", and "target." The **Occupation/Camps** topic appears to relate primarily to nonviolent, and largely legal, tactics involving the protest camps, and includes words related to the strategy of occupation itself ("camp", "climb", "squat") and words related to the areas that were typically targeted by this strategy in the UK during this time period ("road", "quarri", "twyford", "tunnel", "tarmac"). Finally, **International Terror** appears to capture illegal international activities ("bomb", "murder", "kill") involving environmental and leftist extremists ("black", "panther", "shepherd", "ship") and their aftermath ("prison", "sentenc", "jail", "fbi", "trial"). As such, we believe that three of these four protest topics clearly encompass protest activities that can be classified as employing violence or illegal destruction of property: **Violent Protest**, **Direct Action/Ecotage**, and **International Terror**, and we hence use associations with these three topics to classify our UK environmental groups as pursuing violent protest activities below.[5]

Before turning to our group classifications, note that the four final topics in Fig 1 appear to relate to more general (environmental) media discourse. Topic 1 is fairly ambiguous and is labeled **Inspirational Language**, as it contains a range of topwords that communicate optimism ("great", "strong", "left") and inclusiveness ("mani", "togeth", "join"). Topic 5: **News and Culture** encompasses current events that are likely of general interest to DoD's UK environmentalist readership, including family-related issues ("men", "women", "school"), sports ("footbal", "game", "team"), and legal/terrorism updates ("terrorist", "intellig", "court"). Topics 7 and 14 are labeled **General Concern** and **Admonishments** and respectively voice (i) overarching environmental concerns for the survival of the planet and (ii) criticisms of those that do not support the (radical) environmentalist viewpoint.[6] Lastly, while we rely on the topwords to define our topics above, note that we also use the STM to identify a sample of highly representative documents for each topic, and have used these sample documents to qualitatively guide our topic interpretations.

Group Classifications

Focusing on the three violent protest tactic-based topics discussed above (**Violent Protest**, **Direct Action/Ecotage**, and **International Terror**), we next evaluate the extent to which these three topics vary systematically in relation to the presence or

[5] While some instances of **Occupation/Camps** may very well be violent or illegal, the topwords found in this topic, as well as our own readings of the documents most associated with this topic, suggest that these strategies are most directly related to peaceful protests in the current corpus, which leads us to avoid treating this topic as an indicator of violent protest activities.

[6] While we remove stopwords during preprocessing, Topic 14 in particular seems to contain a large number of words that could be considered stopwords. We refrain from removing these additional stopwords in a post hoc fashion given that they are not included in the primary English-language stopwords list that is commonly used in STM analyses (e.g., [1, 13, 19]).

absence of specific group occurrences within our associated documents. To do so, we make use of the aforementioned binary group-occurrence STM covariates, which indicate whether (or not) a group occurred (i.e., was mentioned) within a given document (i.e., sentence sequence). We specifically estimate the effect of a 0-to-1 change in each group's presence in a document on that document's degree of attention to each of our three violent protest-based topics, along with the 95% confidence intervals for this effect. If we find that a given group is positively and significantly associated with at least one of our three violent protest topics, then we classify that group as a violent group. All remaining groups were then classified as nonviolent, as these groups either were not positively and significantly related to *any* of the three topics of interest, or were not sufficiently mentioned across the text of DoD corpus itself—which we take as secondary evidence that a group was nonviolent, given the extremist nature of the DoD publication. We discuss three examples of our estimated quantities below, before summarizing our broader classifications of UK environmental groups[7] as either pursuing or not pursuing violent protest tactics.

Fig 2 presents three examples of our STM's estimated effects—which correspond to three specific environmental groups—to illustrate our classification approach. Turning first to the Anarchist Black Cross subfigure in Fig 2, we find that this group is positively associated with the use of **International Terror** tactics, but not significantly associated with tactics of **Direct Action/Ecotage** or **Violent Protest**. We hence would classify this group as a violent environmental group within our sample given its positive association with at least one of our three violent protest activity topics (i.e., **International Terror**). Similarly, the Reclaim the Streets subfigure in Fig 2 demonstrates that a 0-to-1 change in the occurrence of this group within a given document is significantly and positively associated with **Violent Protest** and **Direct Action/Ecotage**—though not with the **International Terror** topic. Here again we would classify Reclaim the Streets as a violent group based upon the two former positive associations. Finally, the Avon Gorge EF! subfigure in Fig 2 illustrates a group that is found to not exhibit positive associations with any of the three violent protest topics. Avon Gorge EF! would hence be an example of a group that we would not classify as violent for our sample.

After iterating through all of the UK environmental groups within our occurrence indicator sample, we identify a total of 19 groups as having a significant positive association with at least one of our three violent protest activity topics. We thus classify each of these UK environmental groups—listed in Table 1—as violent environmental groups. The remaining groups within our occurrence indicator sample were found to have nonsignificant or negative associations with each of the three identified violent protest topics and were therefore classified as nonviolent. We list these nonviolent groups in Table 2 below. Note that we then combine these latter groups with the remaining groups in our sample—i.e., with those UK environmental groups that were listed within DoD's contact lists but did not have sufficient representation across the DoD texts—to construct our full list of nonviolent UK environmental groups. As mentioned above, we interpret the nonoccurrence of a group within the DoD text itself as an indicator of a group being nonviolent, rather than violent, given the DoD publication's routine and systematic coverage of violent environmental protest activities within the UK during this period.

[7] I.e., among those groups with at least one occurrence across the texts of the DoD corpus.

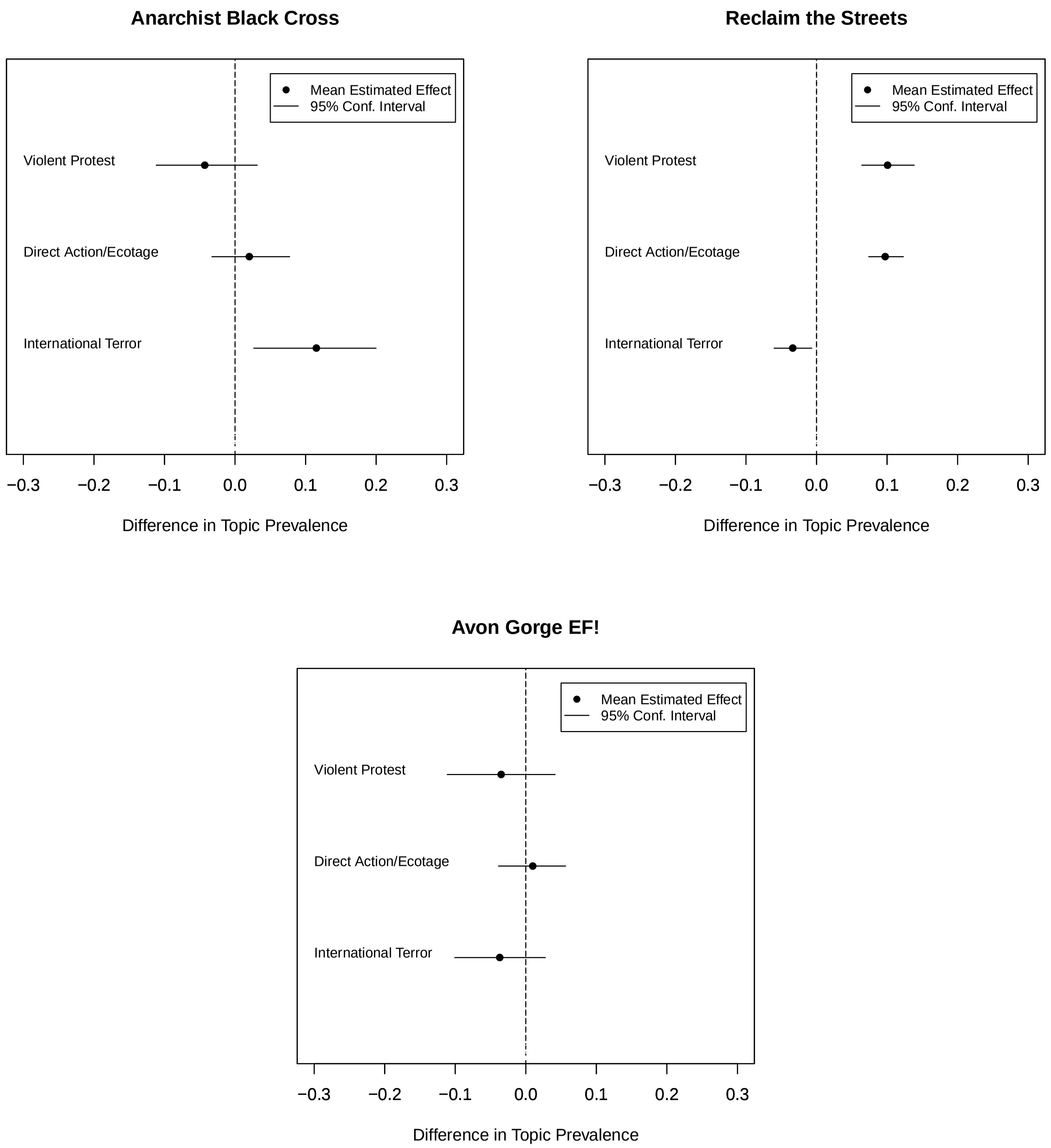

Fig 2. Estimated Associations Between Tactics and Group Ties

Violent Groups
Anarchist Black Cross
Campaign Against The Arms Trade
Cardiff EF!
Glasgow EF!
Haringey Solidarity Group
Hunt Saboteurs Association
Leeds EF!
London GreenPeace
Lune EF!
Manchester EF!
No Opencast
Reclaim the Streets
Reclaim the Valleys
Rising Tide
Road Alert!
South Somerset EF!
The Land is Ours
Tyneside Action for People and Planet
York EF!

Table 1. UK Environmental Groups Associated with Violent and/or Illegal Protest Activities.

Nonviolent Groups		
1 in 12 Club	Friends of People Close to Nature	Primal Seeds
5th May Group	Forest Action Network	Radical Routes
56a Infoshop	Genetic Engineering Network	Save the Hillgrove Cats
Advisory Service for Squatters	Gwynedd and Mon EF!	Simon Jones Memorial Campaign
ALF Supporters Group	Head State Support Group	Solidarity South Pacific
Anarchist Federation	Industrial Workers of the World	South Downs EF!
Anarchist Teapot Action Kitchen	Kate Sharpley Library	Stop Huntingdon Animal Cruelty
Anarchist Youth Network	Kebele Community Centre	TAPOL
Autonomous Centre of Edinburgh	LEAF	The Ecologist
Avon Gorge EF!	Legal Defence and Monitoring Group	The Campaign to Free Vanunu
Bath EF!	London Animal Action	Third Battle of Newbury
CAGE	London Reclaim the Streets	Trident Ploughshares 2000
Campaign Against Runway 2	Liverpool EF!	Undercurrents
Cardigan Bay EF!	Norfolk EF!	Upper Nene EF!
Class War	Nottingham EF!	Wild Things
Cheltenham EF!	OPM Support Group	Woodland Awareness and Defence
Chiapas Link	Oxford EF!	Worthing Anarchist Teapot
East Devon EF!	PaRTiZans	
Faslane Peace Camp	Peat Alert!	

Table 2. UK Environmental Groups *Not* Associated with Violent and/or Illegal Protest Activities.

Extended Descriptives of the Topological and Geographic Characteristics of the Network

To compare ideas of geography and network topology Onnela et. al. [21] has introduced a measure of geographic centrality, which represents how close an individual or group is the geographic center of a community or subgroup. Onnela et. al. define physical centrality as the distance ($d_{ij} = \sqrt{(x_i - x_j)^2 + (y_i - y_j)^2}$) between a given node ($i$) and central point ($S$) of a given community ($C$), where the central point of a community is (X_S, Y_S) using $X_S = (1/n_S) \sum_{i \in C_S} x_i$ and $Y_S = (1/n_S) \sum_{i \in C_S} y_i$ and n_S is the number of members (vertices) in the community. Similar to Onnela et. al., we compare geographic centrality to topological centrality (betweenness centrality in this case) by correlating the two measures where we treat the groupings of violent and nonviolent as exogenously defined. We find that these two correlate at approximately 0.06 which implies that—similar to Onnela et. al.—geographic centrality and network centrality are not highly related in this particular application. See Fig 3.

Descriptive Plots for Radical Environmentalist Networks

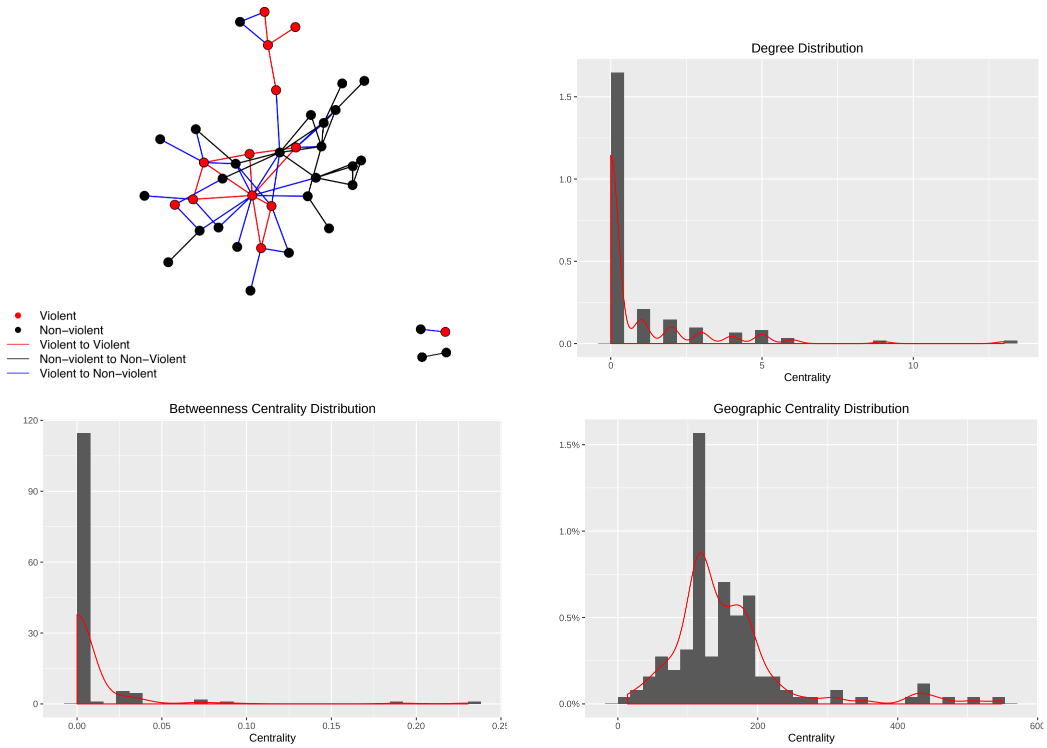

Fig 3. Top Left: Network plot of group to group interaction with isolates not plotted. Top Right: Degree distribution. Bottom Left: Betweenness centrality distribution. Bottom Right: Geographic centrality distribution.

	Nonviolent	Violent
Nonviolent	23.00	26.00
Violent	26.00	13.00

Table 3. Mixing matrix between violent and nonviolent groups for UK Radical groups.

	Nonviolent	Violent
Nonviolent	-0.82	0.92
Violent	0.92	-1.03

Table 4. Z-score matrix between violent and nonviolent groups for UK Radical groups.

Spatial Network Models

Often social networks are represented as a mathematical object known as a *graph* (see for example [22]). A graph can be denoted in either set theoretic notation, where G is a graph which composed of two sets: a vertex set or node set (V) which represents entities such as humans or organizations, and an edge set (E) which represents relations between vertices such as friendship or collaboration. A network or graph can also be represented by a binary adjacency matrix (Y), where y_{ij} is the i to j edge and can take on the value of 0 or 1. If Y is symmetric we say the network is undirected (e.g., $y_{ij} = y_{ji}$) and if Y is not required to be symmetric we say the network is directed (e.g., y_{ij} does not have to equal y_{ji}). Two important measures on a network are the size, $n = |V|$ or $n = |Y|$ and the density, that is defined as the number of observed edges divided by the number of possible edges ($\delta_u = \frac{2|E|}{|V|(|V|-1)}$ and $\delta_d = \frac{|E|}{|V|(|V|-1)}$). Graphs are often visualized for descriptive and interpretative purposes in the social sciences. While the layout is chosen arbitrarily there are some standard choices which accentuate different properties, such as Fruchterman-Reingold (see Fig 4) and geographic layouts (see Fig 4).

A standard network or graph object can be extended to handle spatial locations, we refer to this object as a *spatially embedded network*. The most straightforward way to extend a network to handle spatial information is to follow [23] and take the typical graph, $G = (V, E)$ and create a location function for V, i.e. $\ell : V \to S$, where S is an abstract space. S admits some distance, d (it is not required that this be continuous or a metric) and it can contain social dimensions such as a "Blau" space [23]. For present purposes, take ℓ as given, fixed. Given a network and a location function we can now define a very flexible parametric family of spatial network models, which we call the Inhomogeneous Bernoulli Family.

Inhomogeneous Bernoulli Graph Family

Here, we begin by exploring the *spatial Bernoulli graphs* introduced by [24] and used successfully in the network literature for prediction, and inference in number of different contexts (e.g., [23, 25–29]). This method is one of the most direct ways of incorporating spatial effects into network modeling. This model is a subfamily of the general Exponential Random Graph Models (ERGM) with a spatial attribute [24]. Here, we will focus on the special case were we will assumes that dependence among edges is absorbed by the distance structure – i.e., that edges are conditionally independent. This is similar to temporal models which assume conditional independence by conditioning on the past (e.g, [30, 31]). Following the notation of Butts and others [27, 28, 32], we begin by positing a parametric function, $\mathcal{F}_d(d, \psi)$, that (for some real parameter ψ) maps the distance (d) between two nodes into the probability of an i, j edge. This underlying function is referred to in the literature as the *spatial interaction function* (SIF) and its form governs the relationship between the spatial distribution of nodes and the network structure. (This will be discussed in detail in Section Properties of the

The Six Tie Models with Respect to Distance

Model	$\mathcal{F}_d(d)$
PL	$\frac{p_b}{(1+\alpha d)^\gamma}$
AP	$\frac{p_b}{1+(\alpha d)^\gamma}$
AT	$p_b\left(1-\frac{2}{\pi}\tan^{-1}(\alpha d)\right)$
ED	$\frac{p_b}{e^{\alpha d}}$
LP	$\frac{2p_b}{1+e^{\alpha d}}$
CP	p_b

Table 5. In this article we consider six parametric models from Butts [25] and Butts and Almquist [34].

Spatial Interaction Function.) Thus we define the Inhomogeneous Bernoulli Family model in the following way,

$$\Pr(Y = y \mid D, \psi) = \prod_{\{i,j\}} B\left(y_{ij} \mid \mathcal{F}_d(d_{ij}, \psi)\right), \tag{1}$$

Where $Y \in \{0,1\}^{N\times N}$, $d \in [0,\infty)^{N\times N}$, $\mathcal{F}_d : [0,\infty) \to [0,1]$, B Bernoulli pmf. This model is fundamentally related to the gravity models, i.e., $E[Y_{ij}] = P(i)P(j)F(d_{ij})$, where P is an interaction potential, and F is an impedance or spatial interaction function [33]. The key to these models is the underlying SIF which we will discuss in the next section. Following our discussing of the SIF we will review estimation and simulation from these models and finally conclude by discussing an extension which allows for covariates.

Properties of the Spatial Interaction Function

The SIF is the key component to the inhomogeneous Bernoulli graph models and thus deserves careful discussion. The SIF as presented by Butts [24] is typically modeled via parametric form, where the shape and resulting slopes control the extent to which space models the resulting network structure. In this work we consider six different parametric models for the SIF: (1) Power Law (PL), (2) Attenuated Power Law (ATP), (3) Arctangent Law (AT), (4) Exponential Decay Law (ED), (5) Logistic Probability (LP), and (6) Constant Probability (CP). The formal descriptions can be seen in Table 5. Different SIFs can have strikingly different behavior, for example consider Fig 4 where we have simulated inhomogenious Bernoulli graphs using Butts and Almquist [34] on a spatial grid simulated from a Halton-sequence [32]. The resulting spatial and social network structures are visually different and can result in quite complicated clustering and other interesting topological measures. For a full review of these details see Butts et. al. [27].

Inhomogenious Bernoulli Family Models with Covariates and Statistical Inference

Spiro, Almquist and Butts [28] extended this family of models to handle covariates with a generalized linear model form, and used non-linear optimization to obtain MLE parameters. Spiro, Almquist and Butts [28] define for example the Power Law model with GLM structure as follows,

$$\Pr(Y_{ij} = 1) = \frac{p_{b_{ij}}}{(1+\alpha_{ij} d_{ij})^{\gamma_{ij}}}, \tag{2}$$

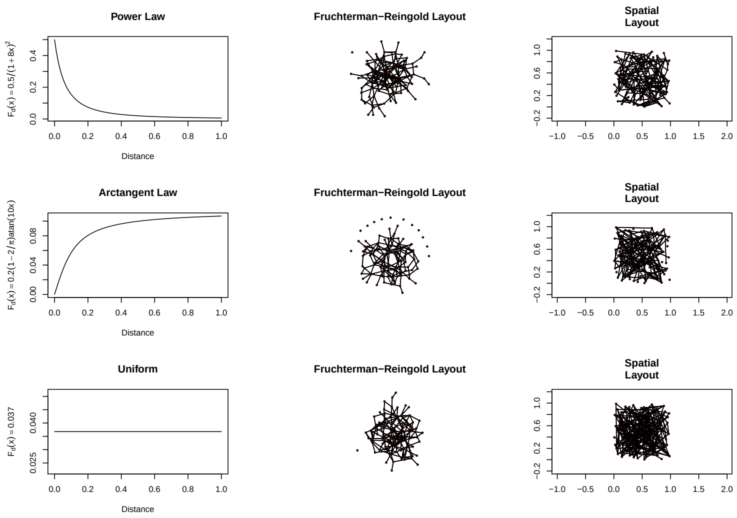

Fig 4. Parametric SIF with resulting spatial networks plotted in standard Fruchterman-Reingold layout and corresponding spatial layout. Networks simulated on on a 100 by 100 grid with spatial locations chosen using a Halton sequence (for details see Almquist and Butts [32]).

where $p_{b_{ij}} = ilogit(\theta * X_{ij})$ and *ilogit* is the inverse logit function, $\alpha_{ij} = \exp(\psi * W_{ij})$, $\gamma_{ij} = exp(\phi * U_{ij})$, and θ, ψ, and ϕ are parameter vectors, and $\mathbf{X}$, $\mathbf{W}$. To fit these models Spiro, Almquist and Butts [28] employ Bayesian point estimation with Importance sampling to fit the exponential family model, for full details see Spiro, Almquist and Butts [28].

Supplement to Spatial Network Model Analysis

To fit the data discussed in the main article we employed the MLE methods of Spiro, Almquist and Butts [28] and use the model selection method of BIC [35] to find the best fitting model. To perform our analysis we begin by modeling the complete network to see which SIF best explains the observed data. The results can be seen in Table 6, where we find that the *arctangent law* SIF is the best fitting model. This corresponds to the following probability function:

$$\Pr(Y_{ij} = 1) = \mathcal{F}_d(x) = p_b \left(1 - \frac{2}{\pi} \tan^{-1}(\alpha x)\right), \quad (3)$$

where $p_{bij} = \text{logit}^{-1}(\theta X_{ij})$, $\alpha_{ij} = \exp(\psi W_{ij})$, θ, ψ are parameter vectors, and X, and W are covariate matrices.

Model Form	BIC
arctangent law	775.981
exponential decay law	776.039
power law	785.207
attenuated power law	785.278

Table 6. BIC Selection table of all considered models. Smallest BIC is chosen as the best fitting model.

To assess the stability of this model, we divided our data into core periods: DoD issues 1-6 in period 1, and issues 7-10 in period 2. We divided into these two time

periods so as to roughly compare the stability of our findings arcos both the pre and post internet sub-periods of our overall sample. We are limited to splitting this into two time periods because of the sparsity of the network(s). We re-ran the spatial network model on each sub-sample, and again obtained the *arctangent law* as the best fitting model on the BIC metric for each time period. The following analysis is similarly robust.

We follow up with a confirmatory model of the *arctangent law*, where we distinguish between the violent and nonviolent organizations and also employ a cosine similarity metric to check if UK environmental organizations' overall levels of shared common interests are driving our findings. In order to hold constant our UK environmental organizations' overall levels of shared common interests, we construct a cosine similarity metric for each unique group-pair (i, j) in our sample. To create our measure, we first classify the documents in our corpus according to their most "dominant topic", $f \in 1 : 15$, based upon our STM's document-indexed posterior word distributions for each topic. We then calculated the portion of a group's total associated documents (x^i_f) that arose from each of our 15 subsets of topic-indexed documents. For each unique group pairing, we next estimated the degree of overlapping topical interest among two groups, i and j, using the approach presented in Fafchamps et al. [36]:

$$w^{ij} = \frac{\sum_f x^i_f x^j_f}{\sqrt{(\sum_f (x^i_f)^2)(\sum_f (x^j_f)^2)}} \tag{4}$$

As such, this measure, w^{ij}, captures the degree of shared common interests among each group pair in our sample, and ranges from 0 (when two groups never appear in documents of the same topic) to 1 (when groups i and j appeared in the same exact topics, at the same proportions). We employ this measure first as a covariate within our *arctangent law* model where we find that the parameter (-18.108 with SE 66.38; p-value = 0.79) is not significant, which led us to ultimately not include this control in our final model. We then fit several models with the violent/nonviolent interaction focusing on a baseline homophily effect in both the probability and scale parameter. We find that there is a significant difference in expected probability of tie between violent and nonviolent groups (the parameter estimates of this resulting model, SE and p-values can be found in Table 7). Specifically we find that (while this is a very sparse network), violent organizations interact heavily at the city level (i.e., relatively short distances), and this decreases rapidly as distance between the groups grow both for within and between tie interaction. We also notice that the nonviolent group is largely a-spatial. These results confirm our second hypothesis, that violent actors are highly localized and likely decentralized in this context. In the main paper we will dissect some these potential effects.

	$\hat{\theta}$	SE	Pvalue
$p_b(violent \leftrightarrow violent)$	-3.54	0.78	0.00*
$p_b(nonviolent \leftrightarrow nonviolent)$	-5.13	0.11	0.00*
$p_b(violent \leftrightarrow nonviolent)$	-4.78	0.35	0.00*
$\alpha(violent \leftrightarrow violent)$	-5.53	1.64	0.00*
$\alpha(nonviolent \leftrightarrow nonviolent)$	-11.39	11.18	0.31
$\alpha(violent \leftrightarrow nonviolent)$	-5.97	0.93	0.00*

Table 7. Parameter table for the SIF for *arctangent law* functional form with homophily terms for violent to violent interaction, nonviolent to nonviolent interaction, and cross group interaction violent to nonviolent (and vice a versa due to the symmetry in the network). * signifies significance at the $\alpha = 0.05$ level.

References

1. Almquist ZW, Bagozzi BE. Using Radical Environmentalist Texts to Uncover Network Structure and Network Features; 2015. Working Paper.

2. Do or Die! Do or Die: Voices from the Ecological Resistance; 2003. Issue Ten.

3. Do or Die! Do or Die: Voices from the Ecological Resistance; 2000. Issue Nine.

4. Eco-Action org. Do or Die: Voices from the Ecological Resistance; Nd. Eco.action.org. ISSN 1462-5989/ISBN 0-9545662-0-3. Accessed on: 07/19/2015. URL: http://www.eco-action.org/dod/index.html.

5. Talon Conspiracy. DO OR DIE - THE COMPLETE SET!; 2014. The Talon Conspiracy. (04/09/14). Accessed on: 07/19/2015. URL: http://thetalonconspiracy.com/2014/04/do-or-die-earth-first/.

6. Barberá P, Bonneau R, Jost JT, Nagler J, Tucker J. Is There Anybody Out There? The Effects of Legislators' Communication with their Constituents; 2014. Paper presented at the ISA Annual Convention.

7. Blaydes L, Grimmer J, McQueen A. Mirrors for Princes and Sultans: Advice on the Art of Governance in the Medieval Christian and Islamic Worlds; 2015. Working Paper. URL: http://aalims.org/uploads/Mirrors%20Princeton.pdf.

8. Brown T. Telling New Stories about our Texts: Next Steps for Topic Modeling in the Humanities; 2012. Presentation. URL: http://mith.umd.edu/topicmodeling/wp-content/uploads/dh2012-slides.pdf.

9. Bagozzi BE, Schrodt PA. The Dimensionality of Political News Reports; 2012. Paper presented at the EPSA Annual Convention.

10. Chen Z, Mukherjee A, Liu B, Hsu M, Castellanos M, Gosh R. Leveraging Multi-Domain Prior Knowledge in Topic Models. Proceedings of the Twenty-Third International Joint Conference on Artificial Intelligence. 2013;.

11. Grimmer J, Stewart BM. Text as Data: The Promise and Pitfalls of Automatic Content Analysis Methods for Political Texts. Political Analysis. 2013;21(3):267–297.

12. Davidov D, Rappoport A, Koppel M. Fully Unsupervised Discovery of Concept-Specific Relationships By Web Mining. Proceedings of the 45th Annual Meeting of the Association of Computational Linguistics. 2007;p. 232–239.

13. Roberts ME, Stewart BM, Tingley D, Lucas C, Leder-Luis J, Gdarian S, et al. Structural Topic Models for Open Ended Responses. American Journal of Political Science. 2014;58(4):172–178.

14. Bagozzi BE, Berliner D. The Politics of Scrutiny in Human Rights Monitoring: Evidence from Structural Topic Models of U.S. State Department Human Rights Reports; 2015. Paper presented at "How Do We Know What We Know? Charting the Future for Human Rights Documentation and Analysis," January 23-24.

15. Bagozzi BE, Berliner D, Almquist ZW. Predicting Government (Non)Responsiveness to Freedom of Information Requests with Supervised Latent Dirichlet Allocation. In: Proceedings of the International Conference on the Advances in Computational Analysis of Political Text - PolText 2016. Dubrovnik, Croatia; 2016. .

16. Quinn KM, Monroe BL, Colaresi M, Crespin MH, Radev DR. How to Analyze Political Attention with Minimal Assumptions and Costs. American Journal of Political Science. 2010;54(1):209–228.

17. Grimmer J. A Bayesian Hierarchical Topic Model for Political Texts: Measuring Expressed Agendas in Senate Press Releases. Political Analysis. 2012;18(1):1–35.

18. Rice D. Measuring the Issue Content of Supreme Court Opinions through Probabalistic Topic Models; 2012. Presented at the 2012 Midwest Political Science Association Conference, Chicago.

19. Roberts ME, Stewart BM. stm-package; 2012. R Documentation.

20. Earth First! Journal. Do or Die Editors Interview; 2006. An interview conducted for the American Earth First! Journal, and made available on eco-action.org. Accessed on: 07/19/2015. URL: http://www.eco-action.org/dod/interview.htm.

21. Onnela JP, Arbesman S, González MC, Barabási AL, Christakis NA. Geographic constraints on social network groups. PLoS one. 2011;6(4):e16939.

22. Wasserman S, Faust K. Social network analysis: Methods and applications. vol. 8. Cambridge university press; 1994.

23. Butts CT, Acton RM. Spatial Modeling of Social Networks. In: Timothy Nyerges RM Helen Couclelis, editor. The Sage Handbook of GIS and Society Research. Thousand Oaks, CA: SAGE Publications; 2011. p. 222–250.

24. Butts CT. Predictability of Large-scale Spatially Embedded Networks. In: Breiger RL, Carley KM, Pattison P, editors. Dynamic Social Network Modeling and Analysis: Workshop Summary and Papers. D.C.: National Academies Press; 2003. p. 313–323.

25. Butts CT. Spatial Models of Large-scale Interpersonal Networks. Carnegie Mellon University: Doctoral Dissertation; 2002.

26. Hipp JR, Perrin AJ. The simultaneous effect of social distance and physical distance on the formation of neighborhood ties. City & Community. 2009;8(1):5–25.

27. Butts CT, Acton RM, Hipp JR, Nagle NN. Geographical Variability and Network Structure. Social Networks. 2012;34:82–100.

28. Spiro ES, Almquist ZW, Butts CT. The Persistence of Division: Geography, Institutions, and Online Friendship Ties. Socius: Sociological Research for a Dynamic World. 2016;2:1–15.

29. Almquist ZW, Butts CT. Predicting Regional Self-Identification from Spatial Network Models. Geographical Analysis. 2015;47(1):50–72.

30. Almquist ZW, Butts CT. Dynamic network logistic regression: A logistic choice analysis of inter-and intra-group blog citation dynamics in the 2004 US presidential election. Political Analysis. 2013;21(4):430–448.

31. Almquist ZW, Butts CT. Logistic network regression for scalable analysis of networks with joint edge/vertex dynamics. Sociological methodology. 2014;44(1):273–321.

32. Almquist ZW, Butts CT. Point process models for household distributions within small areal units. Demographic Research. 2012;26(22):593–632.

33. Haynes KE, Fortheringham AS. Gravity and spatial interaction models. vol. 2 of Scientific Geography. Beverly Hills: Sage publications; 1984.

34. Butts CT, Almquist ZW. networkSpatial: Tools for the Generation and Analysis of Spatially-embedded Networks; 2016. R package version 1.0. Available from: https://r-forge.r-project.org/R/?group_id=2154.

35. Kass RE, Wasserman L. A reference Bayesian test for nested hypotheses and its relationship to the Schwarz criterion. Journal of the american statistical association. 1995;90(431):928–934.

36. Fafchamps M, Leij MJ, Goyal S. Matching and network effects. Journal of the European Economic Association. 2010;8(1):203–231.
